# EEG during dynamic facial emotion processing reveals neural activity patterns associated with autistic traits in children

**DOI:** 10.1093/cercor/bhaf020

**Published:** 2025-02-10

**Authors:** Aron T Hill, Talitha C Ford, Neil W Bailey, Jarrad A G Lum, Felicity J Bigelow, Lindsay M Oberman, Peter G Enticott

**Affiliations:** Cognitive Neuroscience Unit, School of Psychology, Deakin University, Burwood, VIC 3125, Australia; Cognitive Neuroscience Unit, School of Psychology, Deakin University, Burwood, VIC 3125, Australia; Centre for Mental Health and Brain Sciences, Swinburne University of Technology, Hawthorn, VIC 3122, Australia; School of Medicine and Psychology, The Australian National University, Canberra, ACT 2601, Australia; Monarch Research Institute, Monarch Mental Health Group, Sydney, New South Wales 2000, Australia; Cognitive Neuroscience Unit, School of Psychology, Deakin University, Burwood, VIC 3125, Australia; Cognitive Neuroscience Unit, School of Psychology, Deakin University, Burwood, VIC 3125, Australia; Noninvasive Neuromodulation Unit, Experimental Therapeutics and Pathophysiology Branch, National Institute of Mental Health, National Institutes of Health, Bethesda, MD, United States; Cognitive Neuroscience Unit, School of Psychology, Deakin University, Burwood, VIC 3125, Australia

**Keywords:** aperiodic activity, autism, connectivity, electroencephalography, oscillations

## Abstract

Altered brain connectivity and atypical neural oscillations have been observed in autism, yet their relationship with autistic traits in nonclinical populations remains underexplored. Here, we employ electroencephalography to examine functional connectivity, oscillatory power, and broadband aperiodic activity during a dynamic facial emotion processing task in 101 typically developing children aged 4 to 12 years. We investigate associations between these electrophysiological measures of brain dynamics and autistic traits as assessed by the Social Responsiveness Scale, 2nd Edition (SRS-2). Our results revealed that increased facial emotion processing–related connectivity across theta (4 to 7 Hz) and beta (13 to 30 Hz) frequencies correlated positively with higher SRS-2 scores, predominantly in right-lateralized (theta) and bilateral (beta) cortical networks. Additionally, a steeper 1/*f*-like aperiodic slope (spectral exponent) across fronto-central electrodes was associated with higher SRS-2 scores. Greater aperiodic-adjusted theta and alpha oscillatory power further correlated with both higher SRS-2 scores and steeper aperiodic slopes. These findings underscore important links between facial emotion processing-related brain dynamics and autistic traits in typically developing children. Future work could extend these findings to assess these electroencephalography-derived markers as potential mechanisms underlying behavioral difficulties in autism.

## Introduction

The ability to accurately interpret complex emotional information from human faces is essential for successful social interaction, enabling an individual to infer the intentions and emotional states of others (Collin et al. 2013; Calvo and Nummenmaa 2016). Facial emotion recognition is present within the first year of life and continues to develop throughout childhood, reaching maturity in adolescence or early adulthood (Walker-Andrews 1998; Leppänen and Nelson 2008; Meinhardt-Injac et al. 2020) coinciding with the extensive structural and functional changes that take place during these important neurodevelopmental periods (Aylward et al. 2005; Braams and Crone 2017). Facial emotion processing (FEP) difficulties have been consistently associated with autism spectrum disorder (hereafter referred to as *autism*), a heterogenous neurodevelopmental condition that is characterized by impairments in social communication and interaction, restricted and repetitive behaviors, and atypical sensory processing (Uljarevic and Hamilton 2013; Lord et al. 2020). Indeed, FEP impairments may be one of the earliest indicators of abnormal brain development in autism (Dawson et al. 2005), and research has repeatedly shown that the ability to interpret and understand others’ emotions is compromised in autistic individuals (Poljac et al. 2013; Uljarevic and Hamilton 2013), with these difficulties present from early childhood (Rump et al. 2009; Lacroix et al. 2014; Fridenson-Hayo et al. 2016).

Beyond findings specific to autism, several studies have also identified links between FEP abilities and autistic traits in the broader, nonclinical population. This includes longitudinal research indicating that elevated autistic social difficulties in children are associated with poorer facial emotion recognition ability later in adulthood (Reed et al. 2021). Additionally, cross-sectional studies have shown that individuals with higher autistic traits exhibit delayed reaction times in tasks that require them to recognize others’ mental states (Miu et al. 2012) and display subtle difficulties in recognizing emotional facial expressions in partially covered faces (Pazhoohi et al. 2021). Together, these findings broadly align with the clinical literature indicating impairments in FEP in autism (Rump et al. 2009; Uljarevic and Hamilton 2013; Fridenson-Hayo et al. 2016; Loth et al. 2018; Griffiths et al. 2019). What remains less clear, however, are the precise neural mechanisms underlying FEP across nonclinical populations. This gap in understanding can be addressed by employing methods such as electroencephalography (EEG) to record brain activity during FEP tasks. EEG enables noninvasive recording of electrical neural activity produced by the brain using electrodes placed across the scalp and has high temporal resolution (millisecond timescale), and good tolerance to movement, making it well suited to studying developmental populations (Bell and Cuevas 2012; Herve et al. 2022).

In autism, studies of altered neural activity during FEP have predominantly focused on analysis of EEG-derived event-related potentials (ERPs), which represent brain responses both time- and phase-locked to a stimulus (Kappenman and Luck 2012). The most consistent observations have been atypical modulation of the N170 potential, which is sensitive to faces and is theorized to reflect early encoding of facial stimuli (Bentin et al. 1996; Taylor et al. 2004). Specifically, reduced N170 amplitudes and/or delayed N170 latencies have been reported in several studies of autistic children, relative to neurotypical children (de Jong et al. 2008; Batty et al. 2011; Tye et al. 2014), with the N170 latency to upright human faces being accepted into the Food and Drug Administration's Biomarker Quantification Program in 2019 (McPartland et al. 2020).

While ERPs offer valuable insights into time- and phase-locked neural responses to facial stimuli (David et al. 2006; Kappenman and Luck 2012), alternative EEG analysis methods can provide complementary perspectives on brain activity during FEP. For example, leveraging the high temporal resolution of EEG, functional connectivity measures can elucidate the dynamic interactions between neural networks that underpin FEP. Furthermore, examining neural oscillations enables a more detailed understanding of how specific frequency bands contribute to the processing of emotional facial expressions, offering a richer depiction of the underlying neural mechanisms (Cohen and Gulbinaite 2014; Herrmann et al. 2016). Presently, however, few studies have investigated EEG activity during FEP using methods other than ERPs. Among non-ERP studies, reduced frequency delta and theta power has been reported in autistic adults and adolescents (Yang et al. 2011; Tseng et al. 2015), while diminished theta connectivity in response to viewing emotional faces has been reported in autistic children, relative to neurotypical controls (Yeung et al. 2014). Additionally, using magnetoencephalography (MEG) data and taking a whole brain network-based approach, Safar et al. (Safar et al. 2018) found increased alpha-band connectivity in autistic children during processing of emotional (happy) faces, while reduced beta-band connectivity has also been reported in autistic children relative to controls during viewing of emotive faces (Safar et al. 2022). These limited findings suggest disrupted neural communication during FEP in autistic individuals. This observation aligns with more well-established reports of atypical functional connectivity patterns across brain networks in autism, which have been documented across multiple recording modalities including functional magnetic resonance imaging (fMRI) (Di Martino et al. 2013; Holiga et al. 2019; Ilioska et al. 2022), EEG (Murias et al. 2007; Shou et al. 2017; Zeng et al. 2017), and MEG (O'Reilly et al. 2017; Lajiness-O'Neill et al. 2018). However, the overall picture remains complex, with evidence for both hyper- and hypo-connectivity across various brain regions and networks (Uddin et al. 2013; Ilioska et al. 2022) (for recent reviews, see: Hull et al. 2016; O'Reilly et al. 2017; Mehdizadefar et al. 2019).

In addition to capturing neural oscillations, the EEG signal also contains information on broadband aperiodic (ie nonoscillatory) activity (He 2014; Donoghue et al. 2020). This arrhythmic scale-free signal demonstrates a 1/*f*-like spectral slope when examined in the frequency domain, whereby power diminishes exponentially with increasing frequency (He 2014; Brake et al. 2024). Although initially regarded as “neural noise,” broadband aperiodic activity has received growing attention as a physiologically relevant signal and potential marker of brain excitation–inhibition (EI) balance, given its sensitivity to drugs known to modulate excitatory (glutamatergic) and inhibitory (GABAergic) circuits (Gao et al. 2017; Colombo et al. 2019). Specifically, a steeper aperiodic slope (ie larger exponent) has been associated with greater inhibitory tone (E < I), while a flatter slope suggests enhanced excitation (E > I) (Gao et al. 2017; Colombo et al. 2019; Waschke et al. 2021; Chini et al. 2022). We caution, however, that aperiodic activity is an emerging field of inquiry, with further research necessary to explore its relationship to complex EI systems in the brain (Brake et al. 2024). Nevertheless, given that disrupted EI balance remains a leading theory of autism (Rubenstein and Merzenich 2003; Yizhar et al. 2011), characterizing differences in aperiodic slope is likely to be of significant value for establishing unique markers of atypical neural activity in this condition. To our knowledge, no EEG studies have investigated aperiodic activity in relation to autistic traits within nonclinical populations.

In this study, we used EEG recorded during a dynamic FEP paradigm (ie brief video clips of children’s faces) to assess associations between autistic traits and (i) functional network connectivity, (ii) aperiodic activity, and (iii) neural oscillatory power in a cohort of typically developing children spanning early-to-middle childhood. These specific neurophysiological metrics were chosen given previous evidence of their disruption in autistic populations, as well as the putative link between aperiodic activity and EI balance (Murias et al. 2007; Gao et al. 2017; O'Reilly et al. 2017; Chini et al. 2022). We chose to examine dynamic, rather than static, stimuli as dynamic facial stimuli are potentially more sensitive and ecologically valid (Bernstein and Yovel 2015; Zinchenko et al. 2018) producing activation across several brain areas including distributed cortical regions such as the fusiform gyrus, superior temporal sulcus (STS), inferior frontal/temporal gyrus, and visual areas (Kilts et al. 2003; LaBar et al. 2003; Sato et al. 2004; Fox et al. 2009; Sato et al. 2015). Examining these neurophysiological metrics and their potential associations with autistic traits in a nonclinical sample also helps to avoid confounds related to co-occurring clinical diagnoses, which are common in autism (Lai et al. 2019; Bougeard et al. 2021), as well as any possible effects of psychotropic medications that can impact EEG recordings (Aiyer et al. 2016).

We hypothesized that autistic trait scores, as measured using the Social Responsiveness Scale, 2nd Edition (SRS-2), would be associated with all three EEG-derived measures of neural activity—connectivity, aperiodic activity, and oscillatory power. However, due to the limited research in this area and the varied findings regarding EEG brain activity patterns during FEP, we refrained from making any specific directional predictions. Instead, we used data-driven approaches including the network-based statistic (NBS; connectivity) (Zalesky et al. 2010) to identify brain networks associated with autistic traits, and cluster-based permutation analyses (Maris and Oostenveld 2007) to explore brain-wide links between oscillatory power or aperiodic activity and autistic traits.

## Materials and methods

### Participants and procedure

The data analyzed in this study were collected as part of a larger project aimed at exploring cognitive function and electrophysiological activity across early-to-middle childhood (Bigelow et al. 2021, 2022; Hill et al. 2022a; Hill et al. 2023), but the electrophysiological data reported here (EEG recordings during a dynamic FEP task) have not been reported elsewhere. The initial sample included 153 typically developing children, as described by their primary caregiver, who were not diagnosed with any neurological or neurodevelopmental disorder. Out of the sample, 118 participants had complete SRS-2 assessments and task-related EEG recordings. The research received ethical approval from the Deakin University Human Research Ethics Committee (2017–065), while approval to approach public schools was granted by the Victorian Department of Education and Training (2017_003429). Written consent was obtained from the primary caregiver of each child prior to commencement of the study. All EEG data were collected during a single experimental session, which was conducted either at the university laboratory or in a quiet room at the participants’ school. SRS-2 caregiver reports were completed at each participating child’s home and then mailed to the investigators. Details of the experimental protocol were also explained to each child who then agreed to participate. Participant demographics are provided in Table 1.

**Table 1:** Participant demographics.

	Mean	SD	Range
Age (years)	9.78	1.71	4.1 to 12.9
Sex (M:F)	59:42	ND	ND
SRS-2 *T*-score	48.88	8.98	37 to 85
WASI FSIQ	111.95	11.67	79 to 133

ND, no data.

### Assessment of autistic traits

Autistic traits were evaluated using the School-Age version (ages 4 to 18 years) of the SRS-2, a 65-item caregiver report rating scale that measures deficits in social behavior and restricted and repetitive behaviors associated with autism (Constantino and Gruber 2005). The SRS-2 has strong psychometric properties and is one of the most widely used measures for characterizing autism symptoms (Bruni 2014). Results on the SRS-2 are reported as total severity scores, which were converted to *T*-scores (mean = 50, SD = 10), with higher scores indicative of more pronounced autism symptoms. The SRS-2 is composed of a Total Score as well as five subscales reflecting more specific dimensions of autism-related symptoms (Social Awareness, Social Cognition, Social Communication, Social Motivation, and Restricted Interests and Repetitive Behavior). For this study, we used Total *T*-scores, rather than specific subscales, as these represent the most reliable measure of general social difficulties related to autism (Bruni 2014).

### E‌EG acquisition and facial emotion processing task

**Fig. 1:**
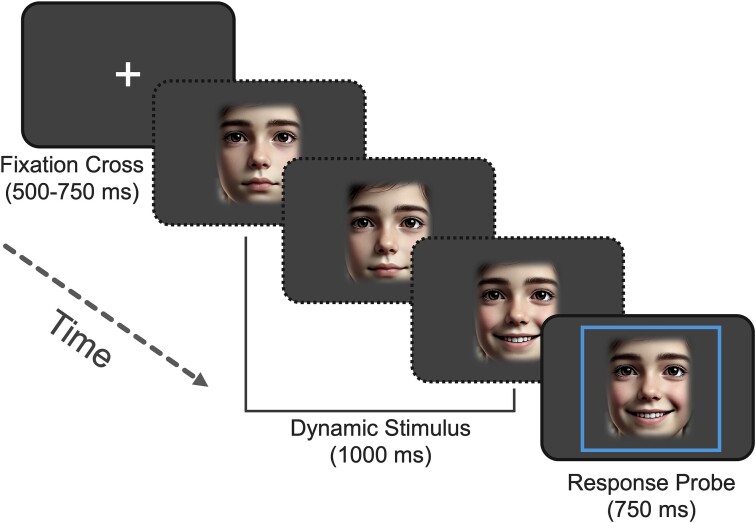
Example single trial of the dynamic facial emotion processing task. A fixation cross is initially presented for between 500 and 750 ms, after which the dynamic facial stimulus is displayed (1,000 ms). The dynamic stimulus period over which the EEG data were analyzed starts as a neutral facial expression, morphing into an emotive expression (happiness or anger) over the course of the presentation period. Immediately following the dynamic stimulus, a square appears (750 ms) around the final frame of the image probing the participant to respond. Note: Face images in this example figure are AI-generated using DALL·E as the CAFE set is copyright-protected.

EEG data were recorded via a 64-channel HydroCel Geodesic Sensor Net (Electrical Geodesics, Inc, USA) containing Ag/AgCl electrodes surrounded by electrolyte-wetted sponges. Recordings were taken in a dimly lit room using NetStation software (version 5.0) via a Net Amps 400 amplifier with a sampling rate of 1 KHz and an online reference at the vertex (Cz electrode). Electrode impedances were checked to ensure they were < 50 KOhms prior to recordings commencing (considered “low” impedance on Geodesic high-input impedance amplifiers). During EEG recordings, participants completed a FEP task (Fig. 1) using stimuli taken from the Child Affective Facial Expression Stimulus Set (CAFE) (LoBue 2014; LoBue and Thrasher 2014). Following presentation of a fixation cross (500 to 750 ms), dynamic (1,000 ms) animated clips of children’s faces expressing either happiness or anger were presented in a randomized order on a 55 cm computer monitor positioned 60 cm from the participant using the E-Prime software (Psychology Software Tools, Pittsburgh, PA). The stimuli began as neutral expressions before dynamically generating each emotional expression. This was achieved using software that dynamically morphed from the neutral to the emotional image over a 1,000 ms time period creating a moving video clip. At the end of each stimulus presentation, a blue box appeared around the final image for 750 ms, signaling the participant to respond with a button press indicating that the subject was feeling either “good,” or “not good.” Timing of the blue box ensured that participant responses did not overlap with the stimulus presentation phase. During the study, participants were also presented with static visual stimuli, which were not analyzed here (see Bigelow et al. 2022 for ERP outcomes reported using static trials).

### E‌EG preprocessing

The EEG data were preprocessed in MATLAB (R2021a; The Mathworks, Massachusetts, USA) using the EEGLAB toolbox (Delorme and Makeig 2004) and custom scripts. The Reduction of Electroencephalographic Artifacts for Juvenile Recordings (RELAX-Jr) software (Hill et al. 2024) was used to clean each EEG file. This automated preprocessing pipeline was adapted from the RELAX software (Bailey et al. 2023) and is specifically optimized for cleaning data recorded in children using Geodesic SenorNet caps. It uses empirical approaches to identify and reduce artifacts within the data, including the use of both multichannel Wiener filters and wavelet-enhanced independent component analysis (ICA). The EEG data were first down-sampled to 500 Hz; then, as part of the RELAX-Jr pipeline, data were bandpass-filtered (0.25 to 80 Hz; fourth-order noncausal zero-phase Butterworth filter). Data were then notch-filtered (47 to 53 Hz; fourth-order noncausal zero-phase Butterworth filter) to remove line noise, and any bad channels were removed using a multistep process that incorporated the “findNoisyChannels” function from the PREP pipeline (Bigdely-Shamlo et al. 2015) as well as multiple outlier detection methods from the original RELAX pipeline (including more than 20% of the electrode time series being affected by extreme absolute amplitudes, extreme amplitude shifts within each 1-s period, and improbable data distributions). Multichannel Wiener filtering (Somers et al. 2018) was used to initially clean blinks, muscle activity, horizontal eye movement, and drift, followed by robust average re-referencing (Bigdely-Shamlo et al. 2015). ICA was then computed using the Preconditioned ICA for Real Data (PICARD) algorithm (Ablin et al. 2018), with the adjusted-ADJUST IC classifier (Leach et al. 2020) used to select components for cleaning using wavelet-enhanced ICA (Castellanos and Makarov 2006). Any electrodes rejected during the cleaning process were then interpolated back into the data using spherical interpolation (mean interpolated channels = 7.59, SD = 3.70). The cleaned data were further segmented from −1 to 1.5 s around the dynamic stimulus onset of which the 0- to 1-s window corresponding to the dynamic stimulus presentation was used in all subsequent analyses.

Any remaining noisy epochs (absolute voltages > 120 μV, or kurtosis/improbable data with standard deviations [SD] > 3 overall, or > 5 at any electrode) were then rejected (mean rejected epochs = 28.82, SD = 22.86 [11.68% of total dataset]). Finally, any participants with < 50 trials were excluded (*n* = 17) to further ensure adequate signal-to-noise ratio and reliability for statistical analyses (Cohen 2014a; Boudewyn et al. 2018). This left a total of *n* = 101 participants with data included in the subsequent analyses (mean number of trials = 80.76, SD = 17.97). Participant demographics are provided in Table 1. Given the relatively wide age and IQ range across participants, we also checked for any possible associations between these values and SRS-2 *T*-scores using Pearson correlations. These revealed no significant associations between age and SRS-2 scores (*r* = −0.082, *P* = 0.415) or IQ and SRS-2 scores (*r* = −0.174, *P* = 0.084).

### Connectivity analysis

Prior to performing the connectivity analyses the estimate of the scalp current density (surface Laplacian) was obtained from the EEG data using the spherical spline method, filtering out spatially broad features of the signal at each electrode and thus better isolating neural activity under each electrode (Perrin et al. 1989; Srinivasan et al. 2007; Cohen 2014b; Carvalhaes and de Barros 2015). This approach is recommended to protect against false-positive inflation of connectivity measurement that can result from volume conduction (Miljevic et al. 2021). Spectral decomposition of the EEG signal was then performed using the Fourier transform with a Hanning window to obtain the complex Fourier coefficients for each subject/electrode/trial across the 1-s window corresponding to the dynamic stimulus. This was done in order to obtain the phase information across different frequencies, which is required for assessment of phase synchronization of the EEG signal between electrodes (Cohen 2014a; Bastos and Schoffelen 2015). Connectivity analysis was then performed across the theta (4 to 7 Hz), alpha (7 to 13 Hz), and beta (13 to 30 Hz) bands using the weighted phase-lag index (wPLI) (Vinck et al. 2011). The wPLI connectivity estimate, combined with the surface Laplacian spatial filter, assisted in reducing the prospect of confounds relating to volume conduction of the signal (Vinck et al. 2011; Tenke and Kayser 2015). The wPLI method disregards instantaneous (ie zero phase-lag) interactions, which are characteristic of volume conduction, thus providing a more accurate connectivity estimate (Vinck et al. 2011; Miljevic et al. 2021).

### Calculation of the aperiodic signal and oscillatory power

To assess aperiodic and oscillatory activity, EEG data were first segmented into 1-s epochs corresponding to the duration of the dynamic stimulus. A Fourier transform (Hanning taper; 1 Hz resolution) was then applied across all channels for each participant to calculate spectral power. The spectral parameterization (*specparam*, formerly *fooof*) toolbox (version 1.0.0) (Donoghue et al. 2020) was then used to parameterize the Fourier transformed EEG data to extract the aperiodic spectral exponent (1/*f-*like broadband slope; frequency range: 1 to 40 Hz) independently for all electrodes. Models were fitted using the “fixed” aperiodic mode, and spectral parameterization settings for the algorithm were: peak width limits = [1, 8], maximum number of peaks = 12, peak threshold = 2, minimum peak height = 0.05. EEG power spectra were also calculated from the same 1-s epoch corresponding to the facial emotion stimulus. The aperiodic activity was then subtracted from the power spectra to leave only the oscillatory (periodic) component of the signal, which was then averaged across the theta (4 to 7 Hz), alpha (7 to 13 Hz), and beta (13 to 30 Hz) bands ready for statistical analysis. This approach was taken to prevent conflating narrowband oscillatory activity with the broadband aperiodic signal (Donoghue et al. 2020; Donoghue et al. 2021; Brake et al. 2024). This was implemented using the Fieldtrip “ft_freqanalysis” function within MATLAB, calling *specparam* functions from the Brainstorm toolbox (Tadel et al. 2011). We applied *specparam* using the same settings for the model as outlined for calculation of the aperiodic signal. Finally, since the calculation of power after spectral parameterization to first remove the aperiodic signal is a relatively new approach, we also ran a traditional (ie nonparameterized) spectral analysis. This involved measuring the absolute EEG power spectra without subtracting the aperiodic activity (ie measuring the combination of periodic and aperiodic activity). Results from these analyses are reported in the Supplementary Materials (Fig. S3) along with grand-average ERPs (Figure S4).

### Statistical analysis

Statistical analyses were performed in R (version 4.0.3; R Core Team 2020) and MATLAB (version 2021a). Correlations between autistic traits (SRS-2 *T*-scores) and functional connectivity for each of the theta, alpha, and beta frequency bands were performed using the NBS MATLAB toolbox (Zalesky et al. 2010), which utilizes nonparametric statistics in order to maintain statistical power while controlling for multiple comparisons (Zalesky et al. 2010). The primary threshold (test-statistic) for electrode pairs was set conservatively (test-statistic: 3.39, equivalent *P*-value of 0.001) to ensure that only robust connectivity differences would be compared at the cluster level and have strict control of Type 1 error (Zalesky et al. 2010; Wang et al. 2023). A value of *P* < 0.05 (two-tailed) was used as the secondary significance threshold for family-wise corrected cluster analysis (5,000 permutations). Subsequent visualization of brain networks was performed using the BrainNet viewer toolbox (Xia et al. 2013).

Associations between SRS-2 *T*-scores and the aperiodic and periodic spectra were examined using nonparametric cluster-based permutation analyses in Fieldtrip using the “ft_freqstatistics” function incorporating the “ft_statfun_correlationT” function with SRS-2 score as the independent variable and the EEG data as the dependent variable (Maris and Oostenveld 2007; Oostenveld et al. 2011). This approach allows for examination of global effects across all electrodes while controlling for multiple comparisons. Being a nonparametric method, it does not depend on the probability distribution of the data making it well suited to EEG data, which often does not meet the assumptions required for parametric analyses (Nichols and Holmes 2002; Maris and Oostenveld 2007). For all comparisons, clusters were defined as more than three neighboring electrodes with a *P*-statistic < 0.05. Monte Carlo p-values (*P* < 0.05, two-tailed) were then subsequently calculated (5,000 iterations). Lastly, we also performed experiment-wise multiple comparison controls using the Benjamini and Hochberg (Benjamini and Hochberg 1995) false discovery rate (reported as *P*_FDR_) across the primary statistical tests comparing connectivity, aperiodic activity, and oscillatory power with SRS-2 scores (total of eight tests).

## Results

### Participant demographics

The proportion of males to females did not differ significantly within the sample, *X*^2^ = 2.86, *P* = 0.097. Welch two-sample *t*-tests also confirmed that there was no difference between males and females in terms of age, *t*(93.00) = −0.39, *P* = 0.70, SRS-2 *T*-score, *t*(87.61) = −0.38, *P* = 0.71, or intellectual function as measured using the Wechsler Abbreviated Scale of Intelligence, Second Edition Full Scale IQ (WASI-FSIQ; conducted in participants aged ≥6 years), *t*(94.79) = − 0.63 *P* = 0.53. Density plots depicting the distribution of SRS-2 scores and age for males and females can be found in the Supplementary Material (Fig. S1).

### Functional connectivity

**Fig. 2:**
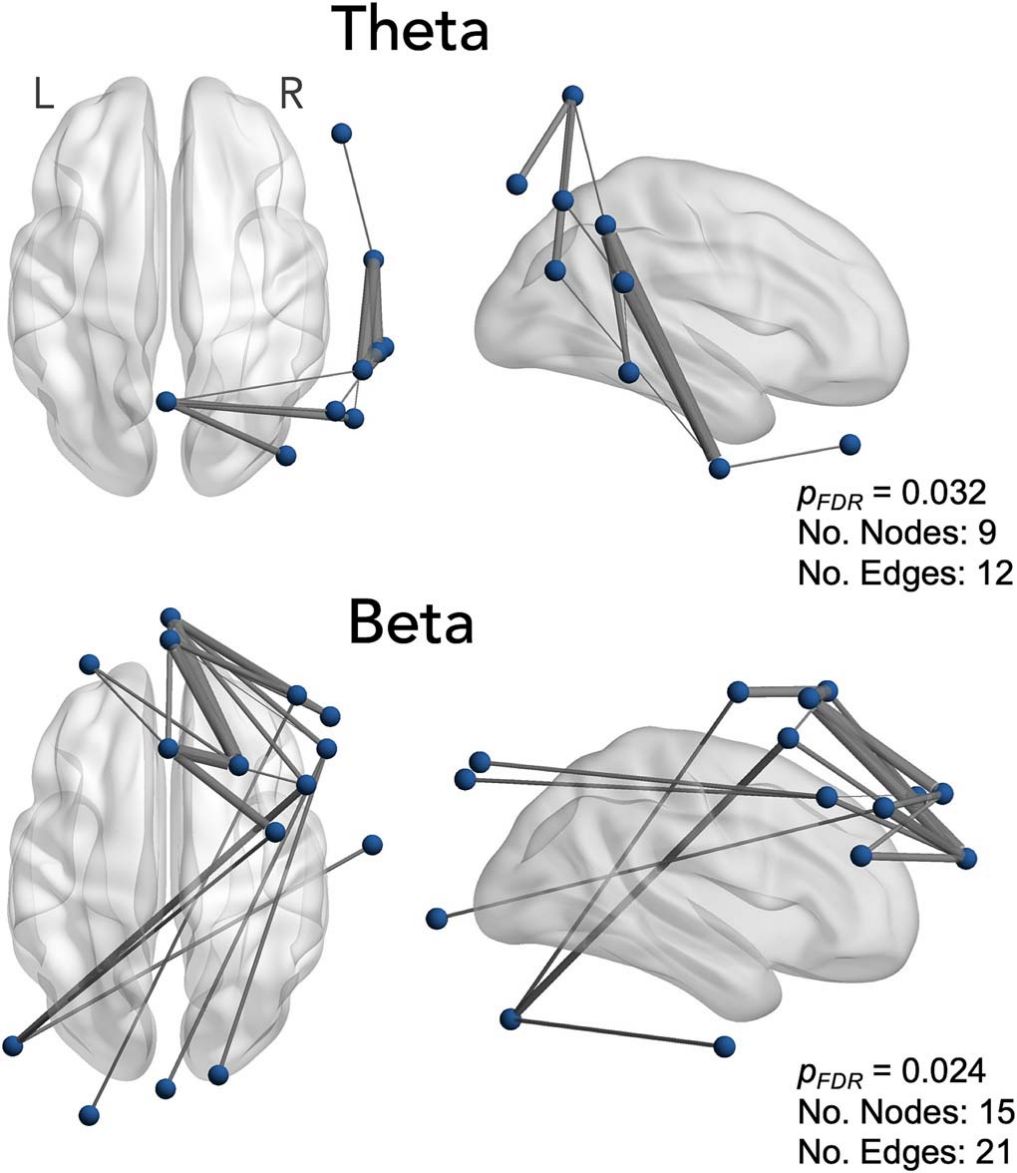
Associations between functional connectivity and level of autistic traits as measured by the social responsiveness scale (SRS-2) total *T*-score. For the theta band, a significant connectivity subnetwork positively associated with the SRS-2 score was identified spanning predominantly right temporo-parietal channels (top). For the beta band, a significant subnetwork positively associated with SRS-2 score was identified spanning both anterior and posterior channels (bottom).

NBS identified subnetworks that showed a significant correlation with autistic traits as measured using SRS-2 *T*-scores across both the theta (*P*_FDR_ = 0.032) and beta (*P*_FDR_ = 0.024) bands (Fig. 2). No significant association was observed between alpha connectivity and autistic traits (*P* > 0.05). The theta subnetwork consisted of nine nodes (electrodes) and 12 edges (connections) spanning predominantly right parieto-temporal cortical regions. The beta subnetwork consisted of 15 nodes and 21 edges spanning bilateral (but predominantly right) frontal, temporal, and posterior regions. Further details, including all electrodes contributing to each subnetwork, are provided in the Supplemental Materials (Fig. S2; Table S1). Next, we ran two additional multiple linear regression models with connectivity (wPLI; averaged across the electrode pairs forming the significant subnetwork from NBS), age, and sex as predictors, and SRS-2 *T*-score as the outcome variable to further assess whether either age or sex could potentially also predict SRS-2 scores. For the theta band, the overall model was significant, *F*(3,97) = 11.84, *P* < 0.001, *R*^2^ = 0.27. Of the predictors, only connectivity was found to significantly contribute to the model, *t*(97) = 5.86, *P* < 0.001 (regression coefficient = 48.58, 95% CI [32.14, 65.02]). For the beta band, the overall model was significant, *F*(3,97) = 19.13, *P* < 0.001, *R*^2^ = 0.37, with only connectivity found to significantly contribute to the model, *t*(97) = 7.49, *P* < 0.001 (regression coefficient = 141.71, 95% CI [104.15, 179.26]).

### Aperiodic slope

**Fig. 3:**
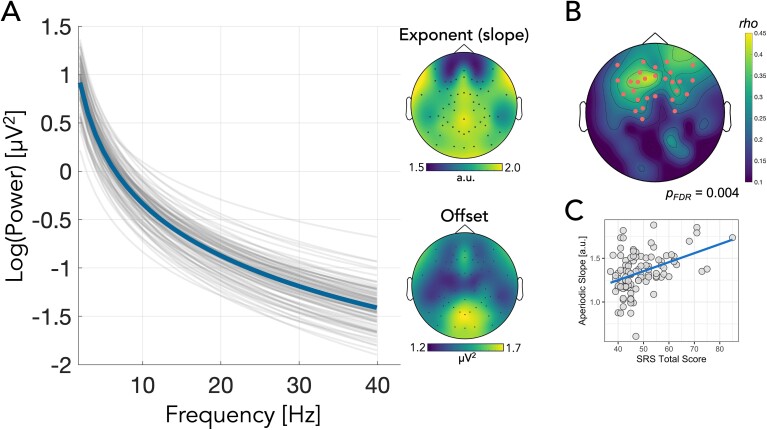
A) The aperiodic exponent (1/*f*-like slope). Thin gray lines represent the individual exponent values for each participant (taken as the average across all EEG channels), while the thick blue line represents the average exponent over all participants. The accompanying topographic maps show the distribution of exponent (top) and offset (bottom) values across the scalp. B) Topographic plot of the spatial distribution of the cluster of electrodes involved in the significant association between aperiodic slope and level of autistic traits as measured by the SRS-2 total *T*-score from the cluster-based permutation analysis. C) Scatterplot showing the association between aperiodic slope taken as an average over the electrodes in the cluster and autistic traits (SRS-2 score).

The spectral parameterisation algorithm performance was assessed using *R*^2^ and error values, taken as an average across all electrodes, to determine the explained variance and error of the model fit relative to the power-frequency spectrum from each participant, respectively. Good model fits to the power-frequency spectrum were observed (mean *R*^2^ = 0.987, SD = 0.005; mean Error = 0.055, SD = 0.016), with all *R*^2^ values above 0.95 (Schaworonkow and Voytek 2021). Cluster-based permutation analyses revealed a positive correlation between aperiodic slope and SRS *T*-scores (*P*_FDR_ = 0.004) indicating steeper slopes in individuals with higher autistic traits. The positive cluster included electrodes spanning bilateral fronto-central regions (Fig. 3; for specific electrodes forming the cluster, see Supplementary Materials Table S1). No significant association was found between SRS-2 *T*-scores and aperiodic offset (*P* > 0.05, two-tailed). Next, as with the connectivity data, we ran additional linear regression models with aperiodic slope (taken as the average across all electrodes forming the significant cluster in the nonparametric permutation-based approach), age, and sex as predictors, and SRS-2 *T*-score as the outcome variable. The overall model was significant, *F*(3,97) = 6.49, *P* < 0.001, *R*^2^ = 0.17. Of the predictors, only aperiodic slope was found to significantly contribute to the model, *t*(97) = 4.30, *P* < 0.001 (regression coefficient = 16.03, 95% CI [8.63, 23.43]).

### Oscillatory power

**Fig. 4:**
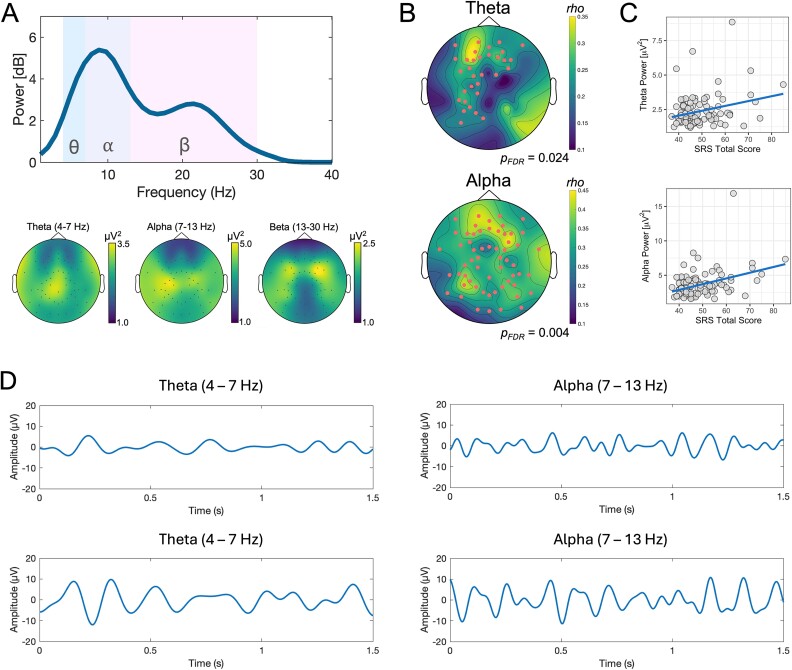
A) Periodic power spectra (ie after spectral parameterization to remove the aperiodic signal) averaged across all electrodes (top) with topographic plots (bottom) showing the distribution of power across the scalp for the theta, alpha, and beta frequencies. B) Topographic plots of the spatial distribution of the electrodes forming the significant cluster for the association between theta (top) and alpha (bottom) power and SRS-2 *T*-score from the cluster-based permutation analysis. C) Scatterplots showing the association between theta (top) and alpha (bottom) power taken as an average over the electrodes in the cluster and autistic traits (SRS-2 score). D) Example EEG time series from two participants in the theta (left; Fz electrode) and alpha (right; Pz electrode) bands. The participant on the top row scored in the low range (bottom quartile for this dataset) for autistic traits (SRS-2 Total T-score = 38) and the participant in the bottom row scored in the high range for this dataset (top quartile; SRS-2 T-score = 71). Prior to creating the plots, the EEG data were bandpass-filtered between 4 and 7 Hz (for theta) and 7 to 13 Hz (for alpha). Visual inspection of this time-domain data indicates generally larger amplitude theta and alpha rhythms in the participant scoring higher on autistic traits.

Cluster-based permutation analyses were run to assess for associations between autistic traits (SRS-2 *T*-score; independent variable) and oscillatory power in each of the theta, alpha, and beta frequency bands (dependent variable). There was a significant positive association between SRS-2 score and power in the theta (*P*_FDR_ = 0.024), and alpha (*P*_FDR_ = 0.004) bands, but not the beta band (*P* > 0.05). The significant cluster within the theta band included predominantly fronto-parietal electrodes, while the alpha cluster incorporated electrodes with a broad scalp distribution, spanning bilateral anterior, central, and posterior regions (Fig. 4; specific electrodes forming the clusters are provided in Supplementary Table S1). As with the aperiodic slope, additional regression analyses were performed with oscillatory power (either theta or alpha; averaged across electrodes within the significant cluster), age, and sex as predictors, and SRS-2 *T*-score as the outcome variable. For the theta frequency, the overall model was significant, *F*(3,97) = 2.951, *P* = 0.036, *R*^2^ = 0.08 with theta power being the only significant predictor contributing to the model, *t*(97) = 2.82 *P* = 0.006 (regression coefficient = 2.31, 95% CI [0.69, 3.94]). For the alpha frequency, the overall model was also significant, *F*(3,97) = 6.20, *P* < 0.001, R^2^ = 0.16, with alpha power being the only significant predictor contributing to the model, *t*(97) = 4.20 *P* < 0.001 (regression coefficient = 1.86, 95% CI [0.98, 2.75]). Finally, cluster-based correlations were also performed using a more traditional approach taking spectral power without parameterization (ie using data containing a combination of periodic and aperiodic activity). These results closely paralleled the findings from the parameterized periodic data (ie significant positive correlations with SRS2-2 *T*-scores), and are reported full in the Supplemental Materials (Fig. S3).

**Fig. 5:**
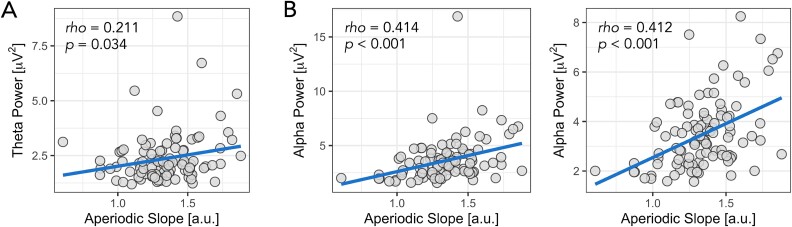
Association between aperiodic-adjusted theta A) and alpha B) power and the aperiodic slope. For alpha power, the plot on the left is the initial correlation, while the plot on the right is after removing the single extreme outlier.

As a final exploratory analysis, we also assessed potential correlations between theta and alpha power and aperiodic slope using the average signal across the electrodes that comprised the significant clusters for each measure. We pursued these additional analyses for two main reasons: (i) recent studies have reported associations between oscillatory power and aperiodic activity (Hill et al. 2022a; Merkin et al. 2023; Manyukhina et al. 2024), prompting us to investigate whether our findings aligned with these observations, and (ii) both alpha (and, to a lesser extent, theta) oscillations and aperiodic slope have been linked to neural inhibitory processes and EI balance (Buzsáki 2002; Haegens et al. 2011; Mathewson et al. 2011; Gao et al. 2017; Manyukhina et al. 2024). For instance, alpha power has been shown to be negatively correlated with Blood Oxygenation Level Dependent (BOLD) fMRI activation, likely reflecting its inhibitory mechanism (Moosmann et al. 2003; Gonçalves et al. 2006), while the aperiodic exponent (spectral slope) has been shown to be modulated by drugs that alter neural inhibition and excitation highlighting its capacity as a putative marker of neuronal EI balance (Gao et al. 2017; Colombo et al. 2019; Waschke et al. 2021). Our results indicated a significant moderate positive correlation (*rho* = 0.414, *P* < 0.001) between alpha power and aperiodic slope. There was also a weak but significant positive association between theta power and aperiodic slope (rho = 0.211, *P* = 0.034). Finally, as there was a single extreme outlier in the alpha power data (z-score > 3.29), we also re-ran the association after its removal; however, the association remained (rho = 0.412, *P* < 0.001). Correlation plots are provided in Fig. 5.

## Discussion

Altered neural activity patterns, including atypical functional connectivity (Coben et al. 2008; Carson et al. 2014; Dickinson et al. 2018; Lajiness-O'Neill et al. 2018) and spectral power (Wang et al. 2013; Neo et al. 2023), have been repeatedly observed in autism. However, the association between neural activity and broader autistic traits in nonclinical populations remains under-investigated. Here, we sought to examine associations between autistic traits measured using the SRS-2- and EEG-derived measures of functional connectivity, spectral power, and aperiodic activity obtained from typically developing children while they were engaged in an ecologically valid social cognitive paradigm (a dynamic FEP task) (Arsalidou et al. 2011; Zinchenko et al. 2018). We found associations between SRS-2 total *T*-scores and EEG activity across all three metrics, highlighting important links between task-related brain activity patterns and autistic traits in typically developing children.

### Functional connectivity

We found a positive association between SRS-2 scores and functional connectivity within the theta and beta bands. Specifically, for each frequency, a subnetwork of stronger connectivity was identified to be associated with higher SRS-2 scores. In the theta band, this subnetwork was lateralized to the right hemisphere, incorporating electrodes positioned over parietal, temporal, and frontal regions; while the beta subnetwork was broader and bilaterally distributed, although predominately right-lateralized.

A dominant systems-level theory of autism, informed largely by resting-state fMRI research, suggests that autism is associated with atypical functional connectivity across brain circuits, including both hyper- and hypo-connectivity (Holiga et al. 2019; Ilioska et al. 2022). Interestingly, hyperconnectivity has been observed predominantly across network hubs located over prefrontal and parietal regions, corresponding closely to the central executive and default mode networks (DMNs) (Buckner et al. 2008; Holiga et al. 2019; Ilioska et al. 2022). Hyperconnectivity between the DMN and other areas was also shown to be associated with social impairments (Ilioska et al. 2022). These observations appear to broadly align with our current findings, which identified subnetworks of stronger connectivity extending across electrodes over fronto-parietal regions, as well as broader central, temporal, and occipital areas in typically developing children with higher SRS-2 scores. Thus, our findings generally fit with large-scale resting-state studies in the neuroimaging literature, which indicate disrupted connectivity across regions such as the DMN and fronto-parietal networks in individuals with autism (Hull et al. 2016; Holiga et al. 2019; Ilioska et al. 2022). Nevertheless, it is important to acknowledge the limited spatial resolution of EEG (typically in the order of several centimeters), which restricts its capacity to offer precise insights into brain network activity (Nunez et al. 1994; Ferree et al. 2001). Similarly, although we observed a positive association between autistic trait scores and connectivity during an FEP task, it remains uncertain whether these results are specific to dynamic facial emotion processing. Future research is required to determine the specificity of these findings. Future studies that integrate EEG with fMRI within the same sample could also yield additional valuable information regarding functional connectivity patterns across diverse temporal and spatial scales (Valdes-Sosa et al. 2011).

It is notable that the relationship between autistic traits and connectivity was predominantly observed in right hemispheric subnetworks, especially within the theta band. This finding is broadly consistent with prior fMRI studies, which have reported connectivity irregularities in these regions among individuals with autism (Igelström et al. 2016; Hao et al. 2022). These areas are also significantly involved in social cognition and face processing (Lombardo et al. 2011; Nomi and Uddin 2015). Importantly, the present findings extend these observations beyond autistic populations to a sample of typically developing children showing a broad range of autistic traits. This suggests that the associations between autistic traits and connectivity extend across the broader autism phenotype, indicating that they are not exclusive to clinical populations, such as autism, where altered sensitivity to faces has been established (Monk et al. 2010; Nomi and Uddin 2015). Further, while the majority of past literature has examined spontaneous resting-state network activity, here we provide insight into neural communication during a dynamic FEP task that would be expected to strongly drive social-cognitive circuits within the brain (Fox et al. 2009). Dynamic FEP tasks require interpretation of changing emotional cues and prediction of social intent. Neuroimaging work has shown dynamic FEP tasks to recruit several interconnected neural networks related to emotion processing such as the amygdala (Kilts et al. 2003; LaBar et al. 2003), STS (Trautmann et al. 2009), fusiform gyrus (Zinchenko et al. 2018), and frontal cortices (Trautmann et al. 2009); for detailed reviews, see (Arsalidou et al. 2011; Zinchenko et al. 2018). Our results therefore suggest that subtle differences in functional neural network activity during dynamic FEP are linked to autistic traits in typically developing children. Moreover, the association between right-lateralized significant subnetworks and autistic traits supports hemispheric specialization theories of emotion processing, consistent with neuroimaging findings showing greater right hemisphere activation during emotion processing (Ahern et al. 1991; Sergent et al. 1992; Lindell 2018) (but see also: Fusar-Poli et al. 2009).

The literature on EEG and MEG connectivity patterns in autism presents mixed findings, likely due to variability in experimental methodologies and participant heterogeneity. A systematic review by O’Reilly et al. (O'Reilly et al. 2017) highlighted evidence of long-range hypoconnectivity in autism, particularly in lower-frequency bands, while higher-frequency bands showed a combination of hypo- and hyperconnectivity. However, more recent large-scale studies, such as Garcés et al. (Garces et al. 2022), have failed to confirm significant alterations in connectivity in autism. A resting-state EEG study by Aykan et al. (Aykan et al. 2021) found that right anterior theta connectivity predicted autistic traits, with stronger connectivity correlating with higher Autism Spectrum Quotient (AQ) scores—a result we were able to replicate using SRS-2 scores (Hill et al. 2022b). In this study, we extend these findings, demonstrating that both theta and beta connectivity during an FEP task are associated with SRS-2 scores. Future research could build on these findings by examining connectivity in response to specific emotional stimuli (eg happy, angry, fearful) separately. This might help to provide more nuanced insights into emotion-specific neural mechanisms, an aspect we were unable to explore here due to a limited number of trials for each emotional category. Integrating EEG with more spatially precise neuroimaging such as fMRI might also be beneficial for more precisely localizing network activity linked to autistic traits during dynamic FEP (Turner et al. 2016).

### Aperiodic activity

We found that children with more pronounced autistic trait expression (ie higher SRS-2 scores) displayed steeper aperiodic slopes across fronto-central regions. Several recent pharmaco-EEG studies have demonstrated sensitivity of the aperiodic slope to drugs known to modulate either glutamatergic or GABAergic pathways, highlighting the aperiodic slope as a potential marker of EI balance within neural circuits (Gao et al. 2017; Colombo et al. 2019; Waschke et al. 2021). From an EI perspective, the present results might suggest greater inhibitory tone (E < I) during FEP in children exhibiting higher autistic traits. Interestingly, this result appears to contrast with broader theories of EI imbalance in autism, which propose that increased excitation may be a possible underlying neurobiological mechanism (Rubenstein and Merzenich 2003; Sohal and Rubenstein 2019). One potential explanation for this difference is the variation in neural activity patterns between neurotypical individuals and the pathophysiological brain dynamics observed in those with a clinical diagnosis. Indeed, it has been recently shown that pharmacological challenges with the GABA agonist arbaclofen can produce divergent responses, in terms of aperiodic slope, between neurotypical and autistic individuals, whereby lower doses of arbaclofen can cause the aperiodic slope to become steeper in autistic individuals but elicit either a flatter slope, or no change, in neurotypical individuals (Ellis et al. 2023). It is also possible that alterations in EI systems are likely to show a degree of regional specificity. For example, autism animal models have shown both hyper- and hypo-excitability across various brain circuits (Goncalves et al. 2017; Golden et al. 2018; Antoine et al. 2019). Similarly, in vivo imaging of GABA and glutamate neuro-metabolites in humans using magnetic resonance spectroscopy (MRS) has also produced differing findings across several brain regions, showing both increases and decreases in glutamate (or Glx, a composite of glutamate + glutamine), and either decreases or no change in GABA (Ford and Crewther 2016; Ajram et al. 2019). Future work using high-density EEG recordings or MEG combined with source-localisation approaches could be beneficial for examining aperiodic activity across specific brain regions (Hedrich et al. 2017).

Research into aperiodic activity in autistic populations is limited. Carter-Leno et al. found that steeper aperiodic slopes from EEG at 10 months correlated with higher autism traits (SRS-2 scores) at 36 months but only in children with lower executive attention ability (Carter Leno et al. 2022). Using resting-state MEG recordings, Manyukina et al. (Manyukhina et al. 2022) reported flatter slopes in autistic boys (aged 6 to 15 years) with below-average IQ (IQ < 85) compared to neurotypical controls. However, no differences were observed in children with average IQs, and autistic children with an IQ above 85 tended to have steeper slopes, consistent with our observation of steeper slopes in individuals with higher autistic traits. Further research is needed to explore the mechanisms underlying these findings; however, one possibility, as suggested by Carter-Leno et al. (Carter Leno et al. 2022), is that steeper slopes may indicate a potential homeostatic compensatory mechanism, with enhanced inhibition in response to excessive excitatory activity to maintain stable EI ratios across neural circuits (Chen et al. 2022). Additionally, although aperiodic slope has been shown to reflect pharmacological modulation of EI systems (Gao et al. 2017; Colombo et al. 2019; Waschke et al. 2021), it likely also captures complex neural dynamics influenced by other physiological mechanisms (Brake et al. 2024). Future studies should therefore aim to better identify the primary neural generator(s) of the aperiodic signal.

### Neural oscillations

In addition to aperiodic activity, we also found a positive association between oscillatory power in the theta and alpha bands and SRS-2 scores. The majority of EEG analyses examining oscillatory power have been performed using resting-state paradigms (Neo et al. 2023). Overall, this literature indicates a tendency for reduced alpha power in autism (Neo et al. 2023), with limited evidence for changes in theta frequencies. However, results have shown considerable variability, possibly reflecting the large degree of heterogeneity present in autism, as well as variations in specific analysis methods used (Garces et al. 2022; Bogéa Ribeiro and da Silva Filho 2023).

Task-related alpha oscillations likely reflect regulatory processes involved in inhibiting specific task-irrelevant brain regions (Jensen and Mazaheri 2010; Herrmann et al. 2016). It is worth noting that instead of showing a region-specific relationship between alpha power during the task and autistic traits, our results indicated a broad topographical spread of greater alpha power related to higher SRS-2 scores. Therefore, one interpretation of our present findings is that the alpha-inhibitory mechanism is broadly heightened in individuals with higher autistic traits, perhaps reflecting widespread reductions in cortical processing in response to the dynamic facial stimuli. Somewhat contrastingly, however, Yang et al. (Yang et al. 2011) reported reduced occipital-parietal alpha power in a small sample of five adolescents and young adults with Asperger’s syndrome relative to controls during viewing of static emotive facial expressions (reported as greater event-related alpha desynchronization using a time–frequency-based approach) and interpreted this as a possible indicator of greater voluntary attention during face recognition. However, a later similar study, also in a small sample (*n* = 10 Asperger’s, *n* = 10 controls), reported no differences (Tseng et al. 2015). As our present results revealed enhanced alpha power in children with higher SRS-2 scores, it is possible that this might represent a tendency for reduced attention during the task in individuals with more pronounced autistic traits. Additionally, the association between theta power and SRS-2 scores was more confined to bilateral frontal and left parietal regions. Given the involvement of fronto-parietal networks and theta oscillations in top–down cognitive control (Vuilleumier and Pourtois 2007; Cavanagh and Frank 2014), we conjecture that this association might represent greater cognitive effort in processing the emotional stimuli in individuals with higher autistic traits. We also note that, unlike previous studies, here we examined oscillatory power after first removing the aperiodic component of the signal, thus preventing the possibility of conflating periodic and aperiodic activity (Donoghue et al. 2020; Donoghue et al. 2021), but our results were maintained even when we did not control for the aperiodic activity.

Finally, we also observed positive associations between theta and alpha power and aperiodic slope values corroborating observations from several recent studies also showing relationships between aperiodic slope and periodic power (Muthukumaraswamy and Liley 2018; Hill et al. 2022a; Merkin et al. 2023; Manyukhina et al. 2024). Given that both theta and alpha oscillations as well as steeper aperiodic slopes have been associated with inhibitory processes, we tentatively interpret this association as reflecting a shared underlying mechanism (ie cortical inhibition) contributing to these two EEG-derived metrics (Jensen and Mazaheri 2010; Mathewson et al. 2011; Colgin 2013; Gao et al. 2017; Zhu et al. 2023; Manyukhina et al. 2024). Considering the substantial value of dependable noninvasive markers for neural inhibitory processes in both cognitive and clinical neuroscience, it would be beneficial for future research to delve deeper into these associations and investigate how they respond to pharmacological and device-based interventions. Such analyses would be particularly relevant in autism, which has been strongly linked to alterations in EI balance, with autistic individuals showing changes in both oscillatory power (Neo et al. 2023) and aperiodic slope (Manyukhina et al. 2022).

### Limitations and future directions

There were several limitations in the present study. First, as we combined emotional conditions across the FEP task, we cannot comment on effects as they might relate to the specific emotional valence of the stimuli. However, our primary objective was to investigate EEG activity and its association with autistic traits while individuals were engaged in FEP, rather than attempting to disentangle differences between specific emotions. Importantly, combining data across stimuli enabled us to ensure an adequate number of trials were included for each participant, thus helping to maximize data quality (ie adequate signal-to-noise ratio) and thus help minimize potential for spurious findings (Cohen 2014b). Nevertheless, it remains a possibility that effects might have been driven more strongly by a particular emotional valance. Future studies could further investigate possible associations between autistic traits and specific emotional conditions to further elucidate the effects of specific facial emotions.

Second, we ran our analyses at the electrode level, rather than using a source-localized signal. Thus, our results cannot provide fine-grained information on precise cortical or subcortical regions. As we did not collect structural MRI data for these participants nor individualized spatial coordinates of the electrodes, we chose not to run source estimation of the EEG signal to avoid the reduced reliability of this approach. Future studies employing high-density electrode arrays (≥128 channels) alongside neural signal source localization and/or supplementary fMRI acquisition could be helpful for enhancing spatial precision (Sohrabpour et al. 2015; Turner et al. 2016). Further, given evidence for a degree of lateralization of emotion processing (Stankovic 2021; Palomero-Gallagher and Amunts 2022), future work might also benefit from examining possible interhemispheric differences in neural activity in relation to autistic traits. We also acknowledge that, despite employing robust data-driven analytical methods, the observed associations between SRS scores and neural activity patterns remain correlational. Future research could further supplement these findings by incorporating additional statistical approaches, such as group-based analyses using a median split of the data. Similarly, the incorporation of complementary instruments, such as the Autism Quotient (AQ) and/or the Repetitive Behavior Scale (RBS), in future studies could potentially provide a more nuanced and multidimensional characterization of autistic traits. Additionally, integrating valence ratings of the dynamic stimuli may also offer deeper insights into how individual differences in emotional and cognitive processing relate to network features.

We also acknowledge that, although we analyzed neural activity while participants were not required to respond (ie they were merely observing the dynamic facial stimuli), it is possible that they were still internally making decisions about how they might respond. This could have influenced our findings. Finally, we also note that while this was a typically developing population (ie participants were described as typically developing by their primary caregiver), it nevertheless still included some participants who obtained SRS-2 scores indicative of a degree of deficiency in social functioning. Given this, in addition to the developmental nature of the cohort, we therefore cannot rule out the possibility that some participants might have later received a diagnosis of autism. However, having a broad range of SRS-2 scores is also a possible strength, as it captures a wider range of social differences.

## Conclusion

Using EEG activity recorded during a dynamic FEP task, we have demonstrated associations between functional connectivity, as well as periodic and aperiodic neural activity and autistic traits in typically developing children spanning early to middle childhood. These findings complement past research examining functional brain processes in autism while extending these findings to a nonclinical sample. Collectively, these results highlight important connections between neural activity patterns and autism traits that extend to the broader population of typically developing children. These results also provide directions for future research to further elucidate the role of neurophysiological processes in autism, which includes the search for reliable biomarkers to gauge therapeutic outcomes and the detection of mechanisms that might further our understanding of autism pathophysiology.

## Supplementary Material

Supplementary_Materials_bhaf020

## References

[ref1] Ablin P, Cardoso JF, Gramfort A. Faster independent component analysis by preconditioning with hessian approximations. IEEE Trans Signal Process. 2018:66:4040–4049. 10.1109/TSP.2018.2844203.

[ref2] Ahern GL et al. Right hemisphere advantage for evaluating emotional facial expressions. Cortex. 1991:27:193–202. 10.1016/S0010-9452(13)80123-2. 1879148

[ref3] Aiyer R, Novakovic V, Barkin RL. A systematic review on the impact of psychotropic drugs on electroencephalogram waveforms in psychiatry. Postgrad Med. 2016:128:656–664. 10.1080/00325481.2016.1218261. 27467441

[ref4] Ajram LA et al. The contribution of [1H] magnetic resonance spectroscopy to the study of excitation-inhibition in autism. Prog Neuro-Psychopharmacol Biol Psychiatry. 2019:89:236–244. 10.1016/j.pnpbp.2018.09.010. 30248378

[ref5] Antoine MW, Langberg T, Schnepel P, Feldman DE. Increased excitation-inhibition ratio stabilizes synapse and circuit excitability in four autism mouse models. Neuron. 2019:101:648–661.e4. 10.1016/j.neuron.2018.12.026. 30679017 PMC6733271

[ref6] Arsalidou M, Morris D, Taylor MJ. Converging evidence for the advantage of dynamic facial expressions. Brain Topogr. 2011:24:149–163. 10.1007/s10548-011-0171-4. 21350872

[ref7] Aykan S et al. Right anterior theta Hypersynchrony as a quantitative measure associated with autistic traits and K-Cl cotransporter KCC2 polymorphism. J Autism Dev Disord. 2021:52:61–72. 10.1007/s10803-021-04924-x. 33635423

[ref8] Aylward EH et al. Brain activation during face perception: evidence of a developmental change. J Cogn Neurosci. 2005:17:308–319. 10.1162/0898929053124884. 15811242

[ref9] Bailey NW et al. Introducing RELAX: an automated pre-processing pipeline for cleaning EEG data - part 1: algorithm and application to oscillations. Clin Neurophysiol. 2023:149:178–201. 10.1016/j.clinph.2023.01.017. 36822997

[ref10] Bastos AM, Schoffelen JM. A tutorial review of functional connectivity analysis methods and their interpretational pitfalls. Front Syst Neurosci. 2015:9:175. 10.3389/fnsys.2015.00175. 26778976 PMC4705224

[ref11] Batty M, Meaux E, Wittemeyer K, Rogé B, Taylor MJ. Early processing of emotional faces in children with autism: an event-related potential study. J Exp Child Psychol. 2011:109:430–444. 10.1016/j.jecp.2011.02.001. 21458825

[ref12] Bell MA, Cuevas K. Using EEG to study cognitive development: issues and practices. J Cogn Dev. 2012:13:281–294. 10.1080/15248372.2012.691143. 23144592 PMC3491357

[ref13] Benjamini Y, Hochberg Y. Controlling the false discovery rate: a practical and powerful approach to multiple testing. J R Stat Soc Ser B Methodol. 1995:57:289–300. 10.1111/j.2517-6161.1995.tb02031.x.

[ref14] Bentin S, Allison T, Puce A, Perez E, McCarthy G. Electrophysiological studies of face perception in humans. J Cogn Neurosci. 1996:8:551–565. 10.1162/jocn.1996.8.6.551. 20740065 PMC2927138

[ref15] Bernstein M, Yovel G. Two neural pathways of face processing: a critical evaluation of current models. Neurosci Biobehav Rev. 2015:55:536–546. 10.1016/j.neubiorev.2015.06.010. 26067903

[ref16] Bigdely-Shamlo N, Mullen T, Kothe C, Su KM, Robbins KA. The PREP pipeline: standardized preprocessing for large-scale EEG analysis. Front Neuroinform. 2015:9:16. 10.3389/fninf.2015.00016. 26150785 PMC4471356

[ref17] Bigelow FJ, Clark GM, Lum JAG, Enticott PG. The mediating effect of language on the development of cognitive and affective theory of mind. J Exp Child Psychol. 2021:209:105158. 10.1016/j.jecp.2021.105158. 33971552

[ref18] Bigelow FJ, Clark GM, Lum JAG, Enticott PG. Facial emotion processing and language during early-to-middle childhood development: an event related potential study. Dev Cogn Neurosci. 2022:53:101052. 10.1016/j.dcn.2021.101052. 34954666 PMC8717415

[ref19] Bogéa Ribeiro L, da Silva Filho M. Systematic review on EEG analysis to diagnose and treat autism by evaluating functional connectivity and spectral power. Neuropsychiatr Dis Treat. 2023:Volume 19:415–424. 10.2147/ndt.S394363. PMC9968781 36861010

[ref20] Boudewyn MA, Luck SJ, Farrens JL, Kappenman ES. How many trials does it take to get a significant ERP effect? It depends. Psychophysiology. 2018:55:e13049–e13049. 10.1111/psyp.13049. 29266241 PMC5940511

[ref21] Bougeard C, Picarel-Blanchot F, Schmid R, Campbell R, Buitelaar J. Prevalence of autism Spectrum disorder and Co-morbidities in children and adolescents: a systematic literature review. Front Psych. 2021:12:744709. 10.3389/fpsyt.2021.744709. PMC8579007 34777048

[ref22] Braams BR, Crone EA. Longitudinal changes in social brain development: processing outcomes for friend and self. Child Dev. 2017:88:1952–1965. 10.1111/cdev.12665. 27861755

[ref23] Brake N et al. A neurophysiological basis for aperiodic EEG and the background spectral trend. Nat Commun. 2024:15:1514. 10.1038/s41467-024-45922-8. 38374047 PMC10876973

[ref24] Bruni TP . Test review: social responsiveness scale–second edition (SRS-2). J Psychoeduc Assess. 2014:32:365–369. 10.1177/0734282913517525.

[ref25] Buckner RL, Andrews-Hanna JR, Schacter DL. The brain's default network: anatomy, function, and relevance to disease. Ann N Y Acad Sci. 2008:1124:1–38. 10.1196/annals.1440.011. 18400922

[ref26] Buzsáki G . Theta oscillations in the hippocampus. Neuron. 2002:33:325–340. 10.1016/S0896-6273(02)00586-X. 11832222

[ref27] Calvo MG, Nummenmaa L. Perceptual and affective mechanisms in facial expression recognition: an integrative review. Cognit Emot. 2016:30:1081–1106. 10.1080/02699931.2015.1049124. 26212348

[ref28] Carson AM, Salowitz NM, Scheidt RA, Dolan BK, Van Hecke AV. Electroencephalogram coherence in children with and without autism spectrum disorders: decreased interhemispheric connectivity in autism. Autism Res. 2014:7:334–343. 10.1002/aur.1367. 24623657

[ref29] Carter Leno V et al. Infant excitation/inhibition balance interacts with executive attention to predict autistic traits in childhood. Mol Autism. 2022:13:46. 10.1186/s13229-022-00526-1. 36482366 PMC9733024

[ref30] Carvalhaes C, de Barros JA. The surface Laplacian technique in EEG: theory and methods. Int J Psychophysiol. 2015:97:174–188. 10.1016/j.ijpsycho.2015.04.023. 25962714

[ref31] Castellanos NP, Makarov VA. Recovering EEG brain signals: artifact suppression with wavelet enhanced independent component analysis. J Neurosci Methods. 2006:158:300–312. 10.1016/j.jneumeth.2006.05.033. 16828877

[ref32] Cavanagh JF, Frank MJ. Frontal theta as a mechanism for cognitive control. Trends Cogn Sci. 2014:18:414–421. 10.1016/j.tics.2014.04.012. 24835663 PMC4112145

[ref33] Chen L, Li X, Tjia M, Thapliyal S. Homeostatic plasticity and excitation-inhibition balance: the good, the bad, and the ugly. Curr Opin Neurobiol. 2022:75:102553. 10.1016/j.conb.2022.102553. 35594578 PMC9477500

[ref34] Chini M, Pfeffer T, Hanganu-Opatz I. An increase of inhibition drives the developmental decorrelation of neural activity. elife. 2022:11:e78811. 10.7554/eLife.78811. PMC9448324 35975980

[ref35] Coben R, Clarke AR, Hudspeth W, Barry RJ. EEG power and coherence in autistic spectrum disorder. Clin Neurophysiol. 2008:119:1002–1009. 10.1016/j.clinph.2008.01.013. 18331812

[ref36] Cohen MX . Analyzing neural time series data: theory and practice. USA: MIT Press; 2014a.

[ref37] Cohen MX . Introduction to connectivity analysis. In Analyzing neural time series data: theory and practice. USA: MIT Press; 2014b.

[ref38] Cohen MX . Practicalities of EEG measurement and experiment design. In: Analyzing neural time series data: theory and practice. Cambridge, Massachusetts: The MIT Press; 2014c. pp. 61–69.

[ref39] Cohen MX . Surface Laplacian. In: Analyzing neural time series data: theory and practice. USA: MIT Press; 2014d. pp. 275–290.

[ref40] Cohen MX, Gulbinaite R. Five methodological challenges in cognitive electrophysiology. NeuroImage. 2014:85 Pt 2:702–710. 10.1016/j.neuroimage.2013.08.010. 23954489

[ref41] Colgin LL . Mechanisms and functions of theta rhythms. Annu Rev Neurosci. 2013:36:295–312. 10.1146/annurev-neuro-062012-170330. 23724998

[ref42] Collin L, Bindra J, Raju M, Gillberg C, Minnis H. Facial emotion recognition in child psychiatry: a systematic review. Res Dev Disabil. 2013:34:1505–1520. 10.1016/j.ridd.2013.01.008. 23475001

[ref43] Colombo MA et al. The spectral exponent of the resting EEG indexes the presence of consciousness during unresponsiveness induced by propofol, xenon, and ketamine. NeuroImage. 2019:189:631–644. 10.1016/j.neuroimage.2019.01.024. 30639334

[ref44] Constantino JN, Gruber CP. Social responsiveness scale: manual. Los Angeles, CA: Western Psychological Services; 2005.

[ref45] David O, Kilner JM, Friston KJ. Mechanisms of evoked and induced responses in MEG/EEG. NeuroImage. 2006:31:1580–1591. 10.1016/j.neuroimage.2006.02.034. 16632378

[ref46] Dawson G, Webb SJ, McPartland J. Understanding the nature of face processing impairment in autism: insights from Behavioral and electrophysiological studies. Dev Neuropsychol. 2005:27:403–424. 10.1207/s15326942dn2703_6. 15843104

[ref1d] Delorme A. Makeig S. EEGLAB: an open source toolbox for analysis of single-trial EEG dynamics including independent component analysis. J Neurosci Methods. 2004:134:9–21. 10.1016/j.jneumeth.2003.10.009. 15102499

[ref78] de Jong MC, van Engeland H, Kemner C. Attentional effects of gaze shifts are influenced by emotion and spatial frequency, but not in autism. J Am Acad Child Adolesc Psychiatry. 2008:47:443–454. 10.1097/CHI.0b013e31816429a6. 18356706 PMC7616329

[ref47] Di Martino A et al. The autism brain imaging data exchange: towards a large-scale evaluation of the intrinsic brain architecture in autism. Mol Psychiatry. 2013:19:659–667. 10.1038/mp.2013.78. 23774715 PMC4162310

[ref48] Dickinson A et al. Interhemispheric alpha-band hypoconnectivity in children with autism spectrum disorder. Behav Brain Res. 2018:348:227–234. 10.1016/j.bbr.2018.04.026. 29689375 PMC5993636

[ref49] Donoghue T et al. Parameterizing neural power spectra into periodic and aperiodic components. Nat Neurosci. 2020:23:1655–1665. 10.1038/s41593-020-00744-x. 33230329 PMC8106550

[ref50] Donoghue T, Schaworonkow N, Voytek B. Methodological considerations for studying neural oscillations. Eur J Neurosci. 2021:55:3502–3527. 10.1111/ejn.15361. 34268825 PMC8761223

[ref51] Ellis CL et al. The dynamically neurodiverse human brain: measuring excitatory-inhibitory dynamics and identifying homeostatic differences in autistic and non-autistic people, *medRxiv*, 2023.2006.2019.23291507. 2023. 10.1101/2023.06.19.23291507.

[ref52] Ferree TC, Clay MT, Tucker DM. The spatial resolution of scalp EEG. Neurocomputing. 2001:38-40:1209–1216. 10.1016/S0925-2312(01)00568-9.

[ref53] Ford TC, Crewther DP. A comprehensive review of the 1H-MRS metabolite Spectrum in autism Spectrum disorder. Front Mol Neurosci. 2016:9:14. 10.3389/fnmol.2016.00014. PMC4783404 27013964

[ref54] Fox CJ, Iaria G, Barton JJS. Defining the face processing network: optimization of the functional localizer in fMRI. Hum Brain Mapp. 2009:30:1637–1651. 10.1002/hbm.20630. 18661501 PMC6870735

[ref55] Fridenson-Hayo S et al. Basic and complex emotion recognition in children with autism: cross-cultural findings. Mol Autism. 2016:7:52. 10.1186/s13229-016-0113-9. 28018573 PMC5168820

[ref56] Fusar-Poli P et al. Laterality effect on emotional faces processing: ALE meta-analysis of evidence. Neurosci Lett. 2009:452:262–267. 10.1016/j.neulet.2009.01.065. 19348735

[ref57] Gao R, Peterson EJ, Voytek B. Inferring synaptic excitation/inhibition balance from field potentials. NeuroImage. 2017:158:70–78. 10.1016/j.neuroimage.2017.06.078. 28676297

[ref58] Garces P et al. Resting state EEG power spectrum and functional connectivity in autism: a cross-sectional analysis. Mol Autism. 2022:13:22. 10.1186/s13229-022-00500-x. 35585637 PMC9118870

[ref59] Golden CEM, Buxbaum JD, De Rubeis S. Disrupted circuits in mouse models of autism spectrum disorder and intellectual disability. Curr Opin Neurobiol. 2018:48:106–112. 10.1016/j.conb.2017.11.006. 29222989 PMC5825272

[ref60] Gonçalves SI et al. Correlating the alpha rhythm to BOLD using simultaneous EEG/fMRI: inter-subject variability. NeuroImage. 2006:30:203–213. 10.1016/j.neuroimage.2005.09.062. 16290018

[ref61] Goncalves J et al. Testing the excitation/inhibition imbalance hypothesis in a mouse model of the autism spectrum disorder: in vivo neurospectroscopy and molecular evidence for regional phenotypes. Mol Autism. 2017:8:47. 10.1186/s13229-017-0166-4. 28932379 PMC5605987

[ref62] Griffiths S et al. Impaired recognition of basic emotions from facial expressions in young people with autism Spectrum disorder: assessing the importance of expression intensity. J Autism Dev Disord. 2019:49:2768–2778. 10.1007/s10803-017-3091-7. 28361375 PMC6606653

[ref63] Haegens S, Nácher V, Luna R, Romo R, Jensen O. α-Oscillations in the monkey sensorimotor network influence discrimination performance by rhythmical inhibition of neuronal spiking. Proc Natl Acad Sci. 2011:108:19377–19382. 10.1073/pnas.1117190108. 22084106 PMC3228466

[ref64] Hao Z et al. The atypical effective connectivity of right Temporoparietal junction in autism Spectrum disorder: a multi-site study. Front Neurosci. 2022:16:927556. 10.3389/fnins.2022.927556. PMC9340667 35924226

[ref65] He BJ . Scale-free brain activity: past, present, and future. Trends Cogn Sci. 2014:18:480–487. 10.1016/j.tics.2014.04.003. 24788139 PMC4149861

[ref66] Hedrich T, Pellegrino G, Kobayashi E, Lina JM, Grova C. Comparison of the spatial resolution of source imaging techniques in high-density EEG and MEG. NeuroImage. 2017:157:531–544. 10.1016/j.neuroimage.2017.06.022. 28619655

[ref67] Herrmann CS, Struber D, Helfrich RF, Engel AK. EEG oscillations: from correlation to causality. Int J Psychophysiol. 2016:103:12–21. 10.1016/j.ijpsycho.2015.02.003. 25659527

[ref68] Herve E, Mento G, Desnous B, Francois C. Challenges and new perspectives of developmental cognitive EEG studies. NeuroImage. 2022:260:119508. 10.1016/j.neuroimage.2022.119508. 35882267

[ref69] Hill AT, Clark GM, Bigelow FJ, Lum JAG, Enticott PG. Periodic and aperiodic neural activity displays age-dependent changes across early-to-middle childhood. Dev Cogn Neurosci. 2022a:54:101076. 10.1016/j.dcn.2022.101076. 35085871 PMC8800045

[ref70] Hill AT, Van Der Elst J, Bigelow FJ, Lum JAG, Enticott PG. Right anterior theta connectivity predicts autistic social traits in typically developing children. Biol Psychol. 2022b:175:108448. 10.1016/j.biopsycho.2022.108448. 36341882

[ref71] Hill AT et al. EEG microstates in early-to-middle childhood show associations with age, biological sex, and alpha power. Hum Brain Mapp. 2023:44:6484–6498. 10.1002/hbm.26525. 37873867 PMC10681660

[ref72] Hill AT, Enticott PG, Fitzgerald PB, Bailey NW. RELAX-Jr: an automated pre-processing pipeline for developmental EEG recordings, *bioRxiv*, 2024.2004.2002.587846. 2024. 10.1101/2024.04.02.587846. PMC11456615 39370644

[ref73] Holiga Š et al. Patients with autism spectrum disorders display reproducible functional connectivity alterations. Sci Transl Med. 2019:11:eaat9223. 10.1126/scitranslmed.aat9223. 30814340

[ref74] Hull JV et al. Resting-state functional connectivity in autism Spectrum disorders: a review. Front Psych. 2016:7:205. 10.3389/fpsyt.2016.00205. PMC5209637 28101064

[ref75] Igelström KM, Webb TW, Graziano MSA. Functional connectivity between the Temporoparietal cortex and cerebellum in autism Spectrum disorder. Cereb Cortex. 2016:27:bhw079–bh2627. 10.1093/cercor/bhw079. 27073219

[ref76] Ilioska I et al. Connectome-wide mega-analysis reveals robust patterns of atypical functional connectivity in autism. Biol Psychiatry. 2022:94:29–39. 10.1016/j.biopsych.2022.12.018. 36925414

[ref77] Jensen O, Mazaheri A. Shaping functional architecture by oscillatory alpha activity: gating by inhibition. Front Hum Neurosci. 2010:4:186. 10.3389/fnhum.2010.00186. 21119777 PMC2990626

[ref79] Kappenman ES, Luck SJ. ERP components: The ups and downs of brainwave recordings. In: Kappenman ES, Luck SJ, editors. The Oxford handbook of event related potential components. USA: Oxford University Press; 2012.

[ref80] Kilts CD, Egan G, Gideon DA, Ely TD, Hoffman JM. Dissociable neural pathways are involved in the recognition of emotion in static and dynamic facial expressions. NeuroImage. 2003:18:156–168. 10.1006/nimg.2002.1323. 12507452

[ref81] LaBar KS, Crupain MJ, Voyvodic JT, McCarthy G. Dynamic perception of facial affect and identity in the human brain. Cereb Cortex. 2003:13:1023–1033. 10.1093/cercor/13.10.1023. 12967919

[ref82] Lacroix A, Guidetti M, Rogé B, Reilly J. Facial emotion recognition in 4- to 8-year-olds with autism spectrum disorder: a developmental trajectory approach. Res Autism Spectr Disord. 2014:8:1146–1154. 10.1016/j.rasd.2014.05.012.

[ref83] Lai MC et al. Prevalence of co-occurring mental health diagnoses in the autism population: a systematic review and meta-analysis. Lancet Psychiatry. 2019:6:819–829. 10.1016/S2215-0366(19)30289-5. 31447415

[ref84] Lajiness-O'Neill R et al. Patterns of altered neural synchrony in the default mode network in autism spectrum disorder revealed with magnetoencephalography (MEG): relationship to clinical symptomatology. Autism Res. 2018:11:434–449. 10.1002/aur.1908. 29251830

[ref85] Leach SC et al. Adjusting ADJUST: optimizing the ADJUST algorithm for pediatric data using geodesic nets. Psychophysiology. 2020:57:e13566. 10.1111/psyp.13566. 32185818 PMC7402217

[ref86] Leppänen JM, Nelson CA. Tuning the developing brain to social signals of emotions. Nat Rev Neurosci. 2008:10:37–47. 10.1038/nrn2554. 19050711 PMC2976651

[ref87] Lindell A . Chapter 9 - lateralization of the expression of facial emotion in humans. In: Forrester GS, Hopkins WD, Hudry K, Lindell A, editors. Progress in brain research. Vol. 238. Elsevier, Amsterdam, Netherlands; 2018. pp. 249–270. 10.1016/bs.pbr.2018.06.005 30097194

[ref88] LoBue V . 2014. The child affective facial expression (CAFE) set. 10.3389/fpsyg.2014.01532 PMC4285011 25610415

[ref89] LoBue V, Thrasher C. The child affective facial expression (CAFE) set: validity and reliability from untrained adults. Front Psychol. 2014:5:1532. 10.3389/fpsyg.2014.01532. 25610415 PMC4285011

[ref90] Lombardo MV, Chakrabarti B, Bullmore ET, Consortium MA, Baron-Cohen S. Specialization of right temporo-parietal junction for mentalizing and its relation to social impairments in autism. NeuroImage. 2011:56:1832–1838. 10.1016/j.neuroimage.2011.02.067. 21356316

[ref91] Lord C et al. Autism spectrum disorder. Nat Rev Dis Prim. 2020:6:5. 10.1038/s41572-019-0138-4. 31949163 PMC8900942

[ref92] Loth E et al. Facial expression recognition as a candidate marker for autism spectrum disorder: how frequent and severe are deficits? Mol Autism. 2018:9:7. 10.1186/s13229-018-0187-7. 29423133 PMC5791186

[ref93] Manyukhina VO et al. Globally elevated excitation-inhibition ratio in children with autism spectrum disorder and below-average intelligence. Mol Autism. 2022:13:20. 10.1186/s13229-022-00498-2. 35550191 PMC9102291

[ref94] Manyukhina VO, Prokofyev AO, Obukhova TS, Stroganova TA, Orekhova EV. Changes in high-frequency aperiodic 1/f slope and periodic activity reflect post-stimulus functional inhibition in the visual cortex. Imaging Neurosci. 2024:2:1–24. 10.1162/imag_a_00146. PMC12247559 40800396

[ref95] Maris E, Oostenveld R. Nonparametric statistical testing of EEG- and MEG-data. J Neurosci Methods. 2007:164:177–190. 10.1016/j.jneumeth.2007.03.024. 17517438

[ref96] Mathewson KE et al. Pulsed out of awareness: EEG alpha oscillations represent a pulsed-inhibition of ongoing cortical processing. Front Psychol. 2011:2:99. 10.3389/fpsyg.2011.00099. 21779257 PMC3132674

[ref97] McPartland JC et al. The autism biomarkers Consortium for clinical trials (ABC-CT): scientific context, study design, and progress toward biomarker qualification. Front Integr Neurosci. 2020:14:16. 10.3389/fnint.2020.00016. 32346363 PMC7173348

[ref98] Mehdizadefar V, Ghassemi F, Fallah A. Brain connectivity reflected in electroencephalogram coherence in individuals with autism: a meta-analysis. Basic Clin Neurosci. 2019:10:409–417. 10.32598/bcn.9.10.375. 32284830 PMC7149956

[ref99] Meinhardt-Injac B, Kurbel D, Meinhardt G. The coupling between face and emotion recognition from early adolescence to young adulthood. Cogn Dev. 2020:53:100851. 10.1016/j.cogdev.2020.100851.

[ref100] Merkin A et al. Do age-related differences in aperiodic neural activity explain differences in resting EEG alpha? Neurobiol Aging. 2023:121:78–87. 10.1016/j.neurobiolaging.2022.09.003. 36379095

[ref101] Miljevic A, Bailey NW, Vila-Rodriguez F, Herring SE, Fitzgerald PB. EEG-connectivity: a fundamental guide and checklist for optimal study design and evaluation. Biol Psychiatry Cogn Neurosci Neuroimaging. 2021:7:546–554. 10.1016/j.bpsc.2021.10.017. 34740847

[ref102] Miu AC, Pana SE, Avram J. Emotional face processing in neurotypicals with autistic traits: implications for the broad autism phenotype. Psychiatry Res. 2012:198:489–494. 10.1016/j.psychres.2012.01.024. 22425467

[ref103] Monk CS et al. Neural circuitry of emotional face processing in autism spectrum disorders. J Psychiatry Neurosci. 2010:35:105–114. 10.1503/jpn.090085. 20184808 PMC2834792

[ref104] Moosmann M et al. Correlates of alpha rhythm in functional magnetic resonance imaging and near infrared spectroscopy. NeuroImage. 2003:20:145–158. 10.1016/s1053-8119(03)00344-6. 14527577

[ref105] Murias M, Webb SJ, Greenson J, Dawson G. Resting state cortical connectivity reflected in EEG coherence in individuals with autism. Biol Psychiatry. 2007:62:270–273. 10.1016/j.biopsych.2006.11.012. 17336944 PMC2001237

[ref106] Muthukumaraswamy SD, Liley DT. 1/f electrophysiological spectra in resting and drug-induced states can be explained by the dynamics of multiple oscillatory relaxation processes. NeuroImage. 2018:179:582–595. 10.1016/j.neuroimage.2018.06.068. 29959047

[ref107] Neo WS, Foti D, Keehn B, Kelleher B. Resting-state EEG power differences in autism spectrum disorder: a systematic review and meta-analysis. Transl Psychiatry. 2023:13:389. 10.1038/s41398-023-02681-2. PMC10721649 38097538

[ref108] Nichols TE, Holmes AP. Nonparametric permutation tests for functional neuroimaging: a primer with examples. Hum Brain Mapp. 2002:15:1–25. 10.1002/hbm.1058. 11747097 PMC6871862

[ref109] Nomi JS, Uddin LQ. Face processing in autism spectrum disorders: from brain regions to brain networks. Neuropsychologia. 2015:71:201–216. 10.1016/j.neuropsychologia.2015.03.029. 25829246 PMC4506751

[ref110] Nunez PL et al. A theoretical and experimental study of high resolution EEG based on surface Laplacians and cortical imaging. Electroencephalogr Clin Neurophysiol. 1994:90:40–57. 10.1016/0013-4694(94)90112-0. 7509273

[ref111] Oostenveld R, Fries P, Maris E, Schoffelen JM. FieldTrip: open source software for advanced analysis of MEG, EEG, and invasive electrophysiological data. Comput Intell Neurosci. 2011:2011:156869. 10.1155/2011/156869. 21253357 PMC3021840

[ref112] O'Reilly C, Lewis JD, Elsabbagh M. Is functional brain connectivity atypical in autism? A systematic review of EEG and MEG studies. PLoS One. 2017:12:e0175870. 10.1371/journal.pone.0175870. 28467487 PMC5414938

[ref113] Palomero-Gallagher N, Amunts K. A short review on emotion processing: a lateralized network of neuronal networks. Brain Struct Funct. 2022:227:673–684. 10.1007/s00429-021-02331-7. 34216271 PMC8844151

[ref114] Pazhoohi F, Forby L, Kingstone A. Facial masks affect emotion recognition in the general population and individuals with autistic traits. PLoS One. 2021:16:e0257740. 10.1371/journal.pone.0257740. 34591895 PMC8483373

[ref115] Perrin F, Pernier J, Bertrand O, Echallier JF. Spherical splines for scalp potential and current density mapping. Electroencephalogr Clin Neurophysiol. 1989:72:184–187. 10.1016/0013-4694(89)90180-6. 2464490

[ref116] Poljac E, Poljac E, Wagemans J. Reduced accuracy and sensitivity in the perception of emotional facial expressions in individuals with high autism spectrum traits. Autism. 2013:17:668–680. 10.1177/1362361312455703. 22987888

[ref117] R Core Team . R: a language environment for statistical computing. Vienna, Austria: R Foundation for Statistical Computing; 2020.

[ref118] Reed ZE et al. Examining the bidirectional association between emotion recognition and social autistic traits using observational and genetic analyses. J Child Psychol Psychiatry. 2021:62:1330–1338. 10.1111/jcpp.13395. 33655554 PMC8554526

[ref119] Rubenstein JL, Merzenich MM. Model of autism: increased ratio of excitation/inhibition in key neural systems. Genes Brain Behav. 2003:2:255–267. 10.1034/j.1601-183X.2003.00037.x. 14606691 PMC6748642

[ref120] Rump KM, Giovannelli JL, Minshew NJ, Strauss MS. The development of emotion recognition in individuals with autism. Child Dev. 2009:80:1434–1447. 10.1111/j.1467-8624.2009.01343.x. 19765010 PMC3085906

[ref121] Safar K, Wong SM, Leung RC, Dunkley BT, Taylor MJ. Increased functional connectivity during emotional face processing in children with autism Spectrum disorder. Front Hum Neurosci. 2018:12:408. 10.3389/fnhum.2018.00408. 30364114 PMC6191493

[ref122] Safar K et al. Shared and distinct patterns of functional connectivity to emotional faces in autism Spectrum disorder and attention-deficit/hyperactivity disorder children. Front Psychol. 2022:13:826527. 10.3389/fpsyg.2022.826527. PMC8959934 35356352

[ref123] Sato W, Kochiyama T, Yoshikawa S, Naito E, Matsumura M. Enhanced neural activity in response to dynamic facial expressions of emotion: an fMRI study. Brain Res Cogn Brain Res. 2004:20:81–91. 10.1016/j.cogbrainres.2004.01.008. 15130592

[ref124] Sato W, Kochiyama T, Uono S. Spatiotemporal neural network dynamics for the processing of dynamic facial expressions. Sci Rep. 2015:5:12432. 10.1038/srep12432. 26206708 PMC4513292

[ref125] Schaworonkow N, Voytek B. Longitudinal changes in aperiodic and periodic activity in electrophysiological recordings in the first seven months of life. Dev Cogn Neurosci. 2021:47:100895. 10.1016/j.dcn.2020.100895. 33316695 PMC7734223

[ref126] Sergent J, Ohta S, Macdonald B. Functional neuroanatomy of face and object processing. A positron emission tomography study. Brain. 1992:115 Pt 1:15–36. 10.1093/brain/115.1.15. 1559150

[ref127] Shou G et al. Electrophysiological signatures of atypical intrinsic brain connectivity networks in autism. J Neural Eng. 2017:14:046010. 10.1088/1741-2552/aa6b6b. 28540866 PMC5737951

[ref128] Sohal VS, Rubenstein JLR. Excitation-inhibition balance as a framework for investigating mechanisms in neuropsychiatric disorders. Mol Psychiatry. 2019:24:1248–1257. 10.1038/s41380-019-0426-0. 31089192 PMC6742424

[ref129] Sohrabpour A et al. Effect of EEG electrode number on epileptic source localization in pediatric patients. Clin Neurophysiol. 2015:126:472–480. 10.1016/j.clinph.2014.05.038. 25088733 PMC4289666

[ref130] Somers B, Francart T, Bertrand A. A generic EEG artifact removal algorithm based on the multi-channel wiener filter. J Neural Eng. 2018:15:036007. 10.1088/1741-2552/aaac92. 29393057

[ref131] Srinivasan R, Winter WR, Ding J, Nunez PL. EEG and MEG coherence: measures of functional connectivity at distinct spatial scales of neocortical dynamics. J Neurosci Methods. 2007:166:41–52. 10.1016/j.jneumeth.2007.06.026. 17698205 PMC2151962

[ref132] Stankovic M . A conceptual critique of brain lateralization models in emotional face perception: toward a hemispheric functional-equivalence (HFE) model. Int J Psychophysiol. 2021:160:57–70. 10.1016/j.ijpsycho.2020.11.001. 33186657

[ref133] Tadel F, Baillet S, Mosher JC, Pantazis D, Leahy RM. Brainstorm: a user-friendly application for MEG/EEG analysis. Comput Intell Neurosci. 2011:2011:879716. 10.1155/2011/879716. 21584256 PMC3090754

[ref134] Taylor MJ, Batty M, Itier RJ. The faces of development: a review of early face processing over childhood. J Cogn Neurosci. 2004:16:1426–1442. 10.1162/0898929042304732. 15509388

[ref135] Tenke CE, Kayser J. Surface Laplacians (SL) and phase properties of EEG rhythms: simulated generators in a volume-conduction model. Int J Psychophysiol. 2015:97:285–298. 10.1016/j.ijpsycho.2015.05.008. 26004020 PMC4537832

[ref136] Trautmann SA, Fehr T, Herrmann M. Emotions in motion: dynamic compared to static facial expressions of disgust and happiness reveal more widespread emotion-specific activations. Brain Res. 2009:1284:100–115. 10.1016/j.brainres.2009.05.075. 19501062

[ref137] Tseng Y-L, Yang HH, Savostyanov AN, Chien VSC, Liou M. Voluntary attention in Asperger's syndrome: brain electrical oscillation and phase-synchronization during facial emotion recognition. Res Autism Spectr Disord. 2015:13-14:32–51. 10.1016/j.rasd.2015.01.003.

[ref138] Turner BM, Rodriguez CA, Norcia TM, McClure SM, Steyvers M. Why more is better: simultaneous modeling of EEG, fMRI, and behavioral data. NeuroImage. 2016:128:96–115. 10.1016/j.neuroimage.2015.12.030. 26723544

[ref139] Tye C et al. Altered neurophysiological responses to emotional faces discriminate children with ASD, ADHD and ASD+ADHD. Biol Psychol. 2014:103:125–134. 10.1016/j.biopsycho.2014.08.013. 25179537

[ref140] Uddin LQ et al. Salience network-based classification and prediction of symptom severity in children with autism. JAMA Psychiatry. 2013:70:869–879. 10.1001/jamapsychiatry.2013.104. 23803651 PMC3951904

[ref141] Uljarevic M, Hamilton A. Recognition of emotions in autism: a formal meta-analysis. J Autism Dev Disord. 2013:43:1517–1526. 10.1007/s10803-012-1695-5. 23114566

[ref142] Valdes-Sosa PA et al. Multimodal functional network connectivity: an EEG-fMRI fusion in network space. PLoS One. 2011:6:e24642. 10.1371/journal.pone.0024642. 21961040 PMC3178514

[ref143] Vinck M, Oostenveld R, van Wingerden M, Battaglia F, Pennartz CM. An improved index of phase-synchronization for electrophysiological data in the presence of volume-conduction, noise and sample-size bias. NeuroImage. 2011:55:1548–1565. 10.1016/j.neuroimage.2011.01.055. 21276857

[ref144] Vuilleumier P, Pourtois G. Distributed and interactive brain mechanisms during emotion face perception: evidence from functional neuroimaging. Neuropsychologia. 2007:45:174–194. 10.1016/j.neuropsychologia.2006.06.003. 16854439

[ref145] Walker-Andrews AS . Emotions and social development: infants’ recognition of emotions in others. Pediatrics. 1998:102:1268–1271. 10.1542/peds.102.SE1.1268. 9794967

[ref146] Wang J et al. Resting state EEG abnormalities in autism spectrum disorders. J Neurodev Disord. 2013:5:24. 10.1186/1866-1955-5-24. 24040879 PMC3847481

[ref147] Wang X et al. The relationship between disrupted anhedonia-related circuitry and suicidal ideation in major depressive disorder: a network-based analysis. NeuroImage Clin. 2023:40:103512. 10.1016/j.nicl.2023.103512. 37757712 PMC10539666

[ref148] Waschke L et al. Modality-specific tracking of attention and sensory statistics in the human electrophysiological spectral exponent. elife. 2021:10:2021.2001.2013.426522. 10.7554/eLife.70068. PMC8585481 34672259

[ref149] Xia M, Wang J, He Y. BrainNet viewer: a network visualization tool for human brain Connectomics. PLoS One. 2013:8:e68910. 10.1371/journal.pone.0068910. 23861951 PMC3701683

[ref150] Yang HH, Savostyanov AN, Tsai AC, Liou M. Face recognition in Asperger syndrome: a study on EEG spectral power changes. Neurosci Lett. 2011:492:84–88. 10.1016/j.neulet.2011.01.061. 21281694

[ref151] Yeung MK, Han YMY, Sze SL, Chan AS. Altered right frontal cortical connectivity during facial emotion recognition in children with autism spectrum disorders. Res Autism Spectr Disord. 2014:8:1567–1577. 10.1016/j.rasd.2014.08.013.

[ref152] Yizhar O et al. Neocortical excitation/inhibition balance in information processing and social dysfunction. Nature. 2011:477:171–178. 10.1038/nature10360. 21796121 PMC4155501

[ref153] Zalesky A, Fornito A, Bullmore ET. Network-based statistic: identifying differences in brain networks. NeuroImage. 2010:53:1197–1207. 10.1016/j.neuroimage.2010.06.041. 20600983

[ref154] Zeng K et al. Disrupted brain network in children with autism Spectrum disorder. Sci Rep. 2017:7:16253. 10.1038/s41598-017-16440-z. 29176705 PMC5701151

[ref155] Zhu Y et al. Alpha and theta oscillations are causally linked to interference inhibition: evidence from high-definition transcranial alternating current stimulation. Brain Sci. 2023:13. 10.3390/brainsci13071026. PMC10377194 37508958

[ref156] Zinchenko O, Yaple ZA, Arsalidou M. Brain responses to dynamic facial expressions: a normative meta-analysis. Front Hum Neurosci. 2018:12:227. 10.3389/fnhum.2018.00227. PMC5996092 29922137

