# Supplementary material for: EEG during dynamic facial emotion processing reveals neural activity patterns associated with autistic traits in children
Source: Cereb Cortex. 2025 Feb 10;35(2):bhaf020. doi: 10.1093/cercor/bhaf020 (PMC11808805; doi:10.1093/cercor/bhaf020)
Supplement: Supplementary_Materials_bhaf020 [file supplementary_materials_bhaf020.docx]

**Supplementary Materials**

**EEG During Dynamic Facial Emotion Processing Reveals Neural Activity Patterns Associated with Autistic Traits in Children**

Aron T. Hill^1*^, Talitha C. Ford^^1,2^, Neil W. Bailey^^3,4^, Jarrad A. G. Lum^1^, Felicity J. Bigelow^1^, Lindsay M. Oberman^5^, Peter G. Enticott^1^

^These authors contributed equally to the manuscript

1. Cognitive Neuroscience Unit, School of Psychology, Deakin University, Burwood, Australia
2. Centre for Mental Health and Brain Sciences, Swinburne University of Technology, Hawthorn, VIC, Australia
3. School of Medicine and Psychology, The Australian National University, Canberra, ACT, Australia
4. Monarch Research Institute Monarch Mental Health Group, Sydney, New South Wales, Australia
5. Noninvasive Neuromodulation Unit, Experimental Therapeutics and Pathophysiology Branch, National Institute of Mental Health, National Institutes of Health, Bethesda, MD, United States

**Correspondence:**

Aron T. Hill, PhD

Cognitive Neuroscience Unit

School of Psychology

Deakin University

221 Burwood Hwy, Burwood, Victoria, Australia 3125

Ph: +61 3 924 43006

Email: [a.hill@deakin.edu.au](mailto:a.hill@deakin.edu.au)


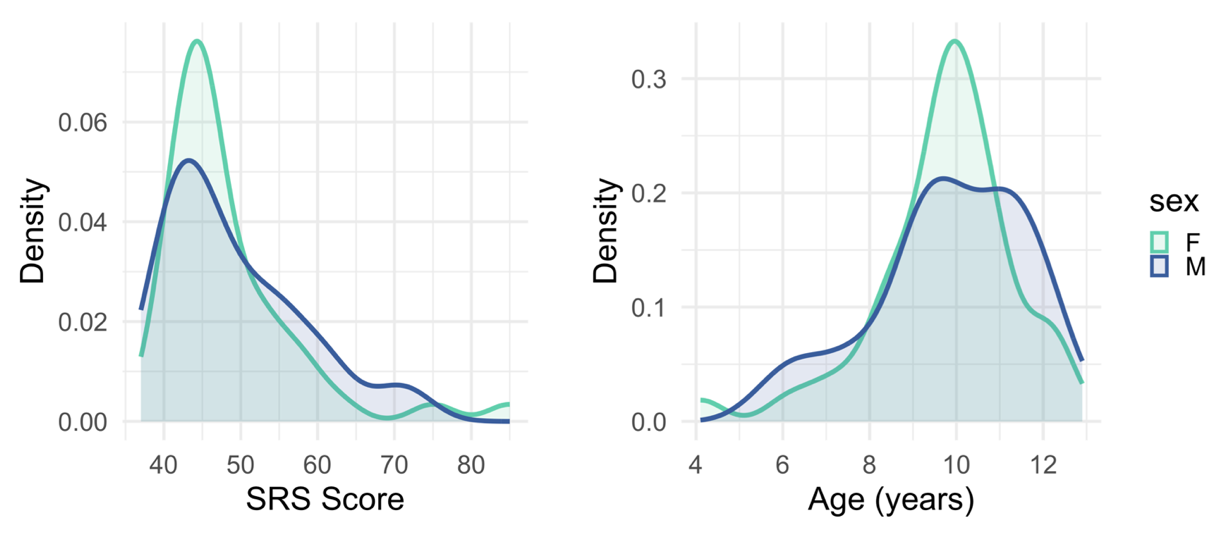


**Figure S1:** Density plot showing the distribution of SRS scores (total t-score; left) and age (right) for females and males. There were no significant differences in SRS score or age between the two sexes (*p* > .05).

**
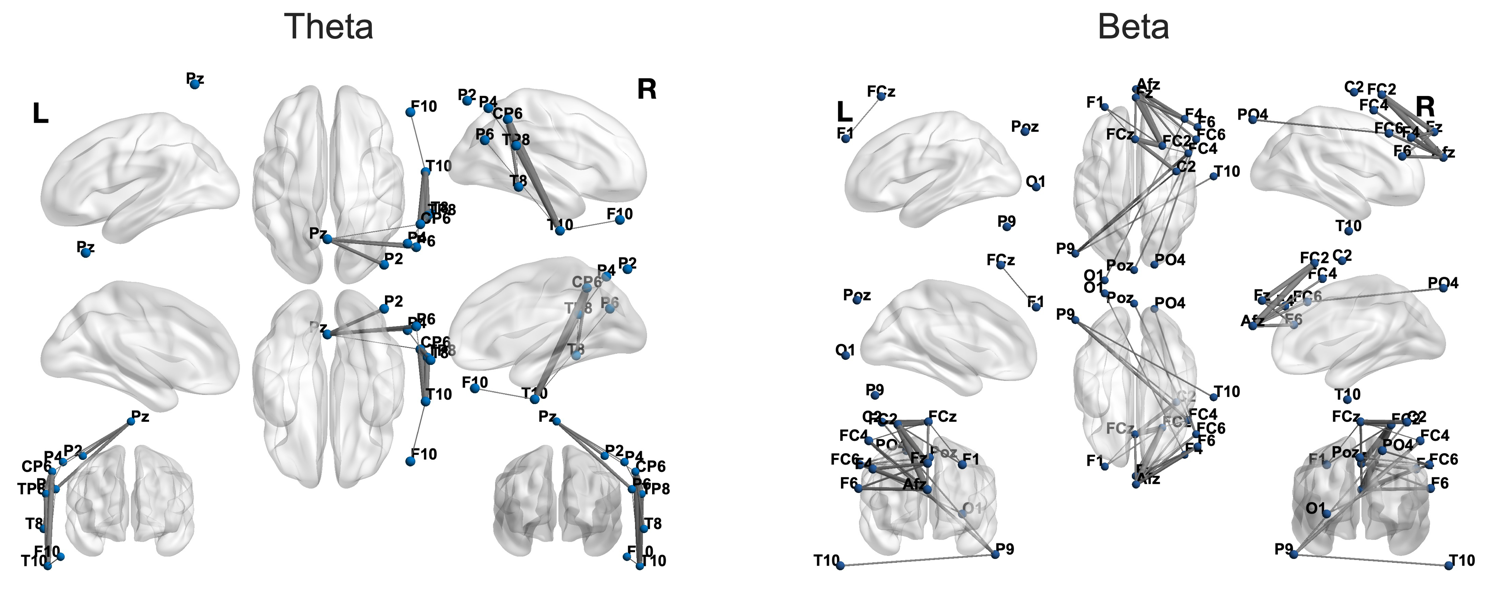
**

**Figure S2:** Plots of the significant subnetworks comparing functional connectivity (wPLI) and autistic traits (SRS-2 total *T*-scores) for the theta (left) and beta (right) frequency bands using the Network Based Statistic.

**
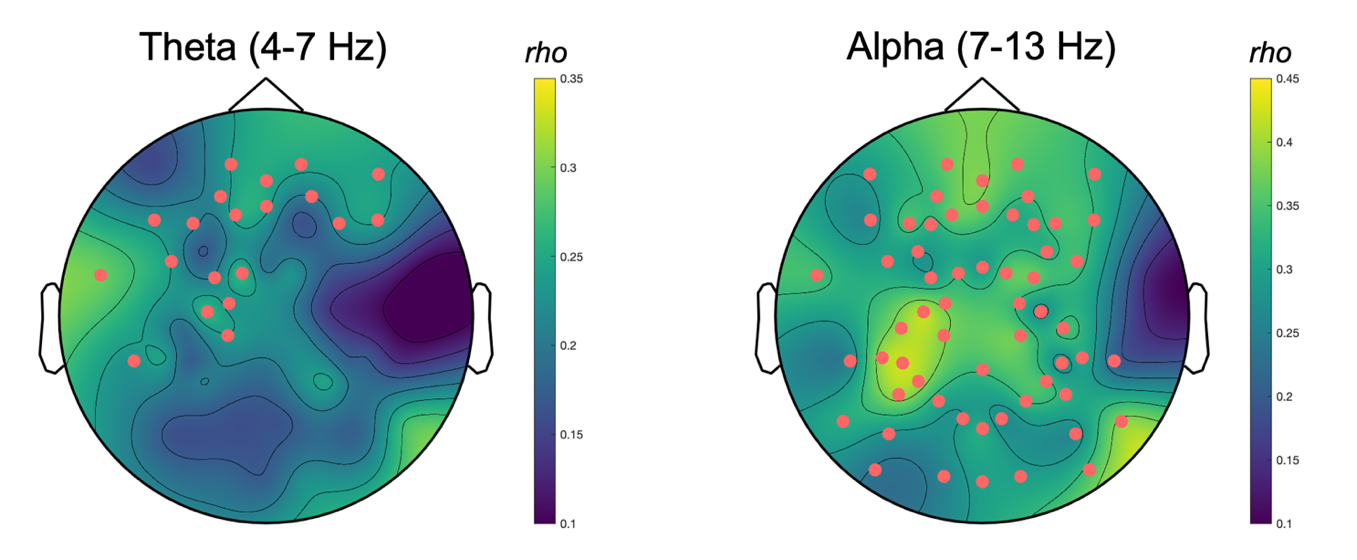
**

**Figure S3:** Topographic plots showing the significant cluster for correlations between total power (i.e., non-parameterised, including both aperiodic and periodic components of the signal) in the theta (4-7 Hz) and alpha (7-13 Hz) bands and SRS *T*-score. Similar to the findings reported in the manuscript, which used a spectral parameterisation approach to account for the aperiodic signal, there were significant positive correlations for both theta (p=0.022) the alpha band (*p* < 0.001) when using this traditional approach.


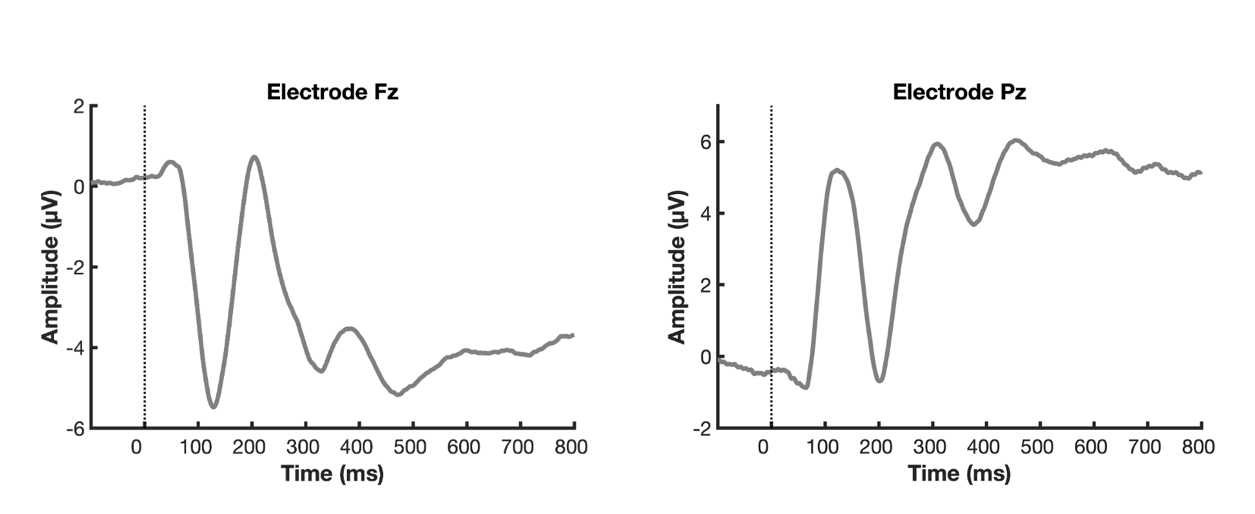


**Figure S4:** Grand average event-related potential (ERP) plots across all participants and trials in response to the dynamic stimuli. ERPs are shown for Anterior (Fz) and Posterior (Pz) electrodes. ERP responses were baseline corrected to the pre-stimulus baseline (-300 to 0 ms).

**Table S1:** Electrodes forming significant clusters (aperiodic activity, oscillation power) or networks (connectivity; electrode pairs) for correlations between EEG activity and SRS-2 *T*-scores.

| **Measure** | **Electrodes** |
| --- | --- |
| Aperiodic Slope | F10, AF4, F2, FCz, Fp2, Fz, FC1, AFz, F1, Fp1, AF3, F3, F5, FC5, FC3, C1, F9, F7, FT7, C3, CP1, C4, FC4, FT8, FC6, F8, F6, F4 |
| Theta Power | F10, AF4, F2, Fp2, Fz, FC1, AFz, F1, Fp1, AF3, F3, F5, FC5, FC3, C1, F9, F7, FT7, C3, CP1, C5, TP7, CP5, P5, P3, P7, P1, PO3, FC2, FC6, F6, F4 |
| Alpha Power | F10, AF4, F2, FCz, Fp2, Fz, FC1, AFz, F1, Fp1, AF3, F3, F5, FC5, FC3, C1, F9, F7, FT7, C3, CP1, C5, T9, T7, TP7, CP5, P5, P3, TP9, P7, P1, PO3, Pz, O1, POz, Oz, PO4, O2, P2, CP2, P4, P10, P8, P6, CP6, TP10, TP8, C6, C4, C2, T8, FC4, FC2, T10, FT8, FC6, F8, F6, F4 |
| Theta Connectivity | Pz-P2, Pz-P4, Pz-P6, Pz-CP6, P4-TP8, CP6-TP8, CP6-T8, TP8-T8, F10-T10, P6-T10, CP6-T10, TP8-T10 |
| Beta Connectivity | FCz-Afz, FCz-F1, FCz-C2, P9-C2, FCz-FC4, Afz-FC4, P9-FC4, FCz-FC2, Fz-FC2, Afz-FC2, F1-FC2, P9-T10, Fz-FC6, Afz-FC6, Poz-FC6, PO4-FC6, Fz-F6, Afz-F6, Fz-F4, Afz-F4, O1-F4 |
